# A shared structure for emotion experiences from narratives, videos, and everyday life

**DOI:** 10.1016/j.isci.2024.110378

**Published:** 2024-06-24

**Authors:** Yanting Han, The COVID-Dynamic Team, Ralph Adolphs

**Affiliations:** 1 Division of Humanities and Social Sciences, California Institute of Technology, Pasadena, CA 91125, USA; 2 Division of Biology and Biological Engineering, California Institute of Technology, Pasadena, CA 91125, USA

**Keywords:** Biological sciences, Neuroscience, Behavioral neuroscience, Cognitive neuroscience

## Abstract

Our knowledge of the diversity and psychological organization of emotion experiences is based primarily on studies that used a single type of stimulus with an often limited set of rating scales and analyses. Here we take a comprehensive data-driven approach. We surveyed 1,000+ participants on a diverse set of ratings of emotion experiences to a validated set of ca. 150 text narratives, a validated set of ca. 1,000 videos, and over 10,000 personal experiences sampled longitudinally in everyday life, permitting a unique comparison. All three types of emotion experiences were characterized by similar dimensional spaces that included valence and arousal, as well as dimensions related to generalizability. Emotion experiences were distributed along continuous gradients, with no clear clusters even for the so-called basic emotions. Individual differences in personality traits were associated with differences in everyday emotion experiences but not with emotions evoked by narratives or videos.

## Highlights

• Surveyed and compared emotion experiences to narratives, videos and real-life events
• Similar low-dimensional spaces for three types of emotion experiences
• Emotion experiences distributed along continuous gradients
• No clear clusters even for the so-called basic emotions

## Graphical abstract

**Figure:**
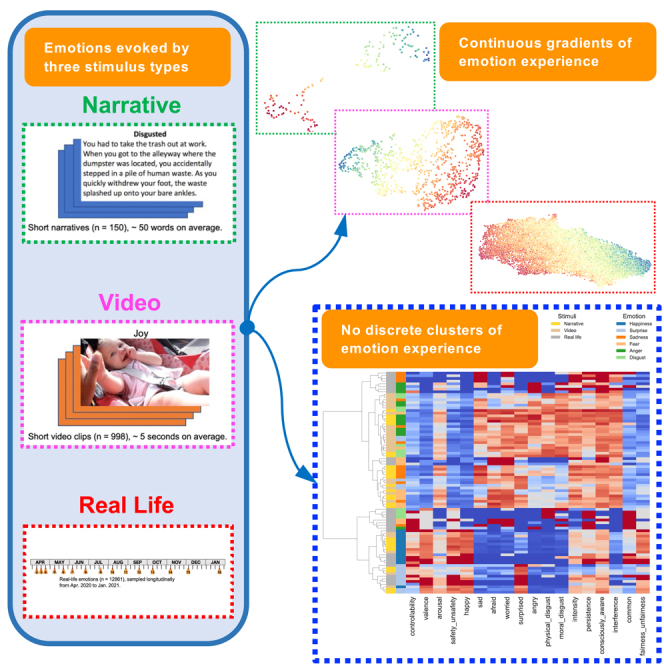

## Introduction

Emotions are ubiquitous,^1^ and their subjective experience is a highly salient aspect of our lives.^2^ Emotions interact with many other psychological processes, such as memory and decision-making, and are a key feature of abnormal functioning in psychiatric disorders.^3^^,^^4^ Despite their patent importance, the scientific understanding of emotions has been modest so far. In good part, this arises from heterogeneity and disagreements in the literature. One likely reason for the disparate findings is that different studies in fact study quite different phenomena (such as concepts, experiences, or effects on cognition), use different analytic approaches and, notably, different stimuli for inducing emotions in the first place. As we review in the following text, each stimulus type in isolation has limitations. We therefore sought to compare emotion experiences evoked, in the same set of participants, across three very different stimulus types: narratives, videos, and everyday life.

One core debate regarding the structure of emotion experience can be roughly summarized as dimensional vs. categorical. The dimensional perspective typically proposes “core affect,” which consists of two dimensions: valence and arousal. Additional components can then be added to account for the full richness and diversity of human emotion experience.^5^^,^^6^^,^^7^ In contrast, classical basic emotion theory argues that emotions are best described by only a few basic and more categorical emotions, such as happiness, surprise, fear, anger, disgust, and sadness.^8^^,^^9^ Recent revised versions of the theory propose a more comprehensive taxonomy, perhaps encompassing around 20 discrete kinds of emotions.^10^ At its core, classical basic emotion theory predicts unique diagnostic patterns of subjective experience, bodily activation, and neural activity for each basic emotion category with consistency and specificity.^11^ The varied evidence for each of these different views comes from studies that have individually used different types of stimuli.

There is a large literature on the self-reported subjective experience of emotion, elicited by a variety of stimuli.^12^ Across the many studies that have used the simplest lexical stimuli, single emotional words, valence and arousal have been identified most consistently as the two dimensions that characterize emotions.^6^ Disagreements on the number and interpretations of dimensions in many cases can be largely attributed to the use of different scales and words.^6^^,^^13^^,^^14^^,^^15^^,^^16^ However, it seems unlikely that reading a single word would induce a potent emotion, and that, instead, participants default to providing what the word is supposed to represent—that is, the word’s conceptual meaning. Therefore, it can be argued that these studies using single words measured the conceptual semantic space of the emotional words, rather than people’s actual emotion experiences. In contrast, narratives provide a more vivid description of an emotional situation and are likely more specific and effective at eliciting emotion experiences.^17^ In the present study, we used one such set that had been used in a prior study to characterize the patterns of evoked brain activations.^18^

More potent inducers of emotion experiences than either words or narratives would be audiovisual stimuli. Videos such as episodes from films are commonly used to elicit emotions but are often limited to a small number of prototypical emotion examples belonging to a few categories.^19^^,^^20^ Moreover, participants are typically asked to rate just the intended emotion categories but not a comprehensive set of scales.^21^^,^^22^^,^^23^^,^^24^ The results thus test primarily for the successful elicitation of *a priori* defined target emotions (as defined by the experimenter) but cannot provide strong evidence one way or the other about the categorical nature of emotion experiences,^25^ since the stimuli lack the number and diversity required. However, there are important exceptions to this. For instance, Cowen and Keltner recently developed a new set of short videos targeting 34 pre-defined emotion categories,^10^ a substantial improvement over previous sets in terms of diversity and quantity of stimuli (there are over 2,000 videos in their database). The present study used, as its second set of stimuli, a selection of these same videos.

Although various kinds of stimuli have been shown to be effective at inducing emotions in the lab, sampling people’s subjective experiences in daily life is of course far less constrained and comes with greater ecological validity.^26^ Prior studies have examined the prevalence of certain emotions in daily life by asking participants to indicate the presence of emotion categories and found that, in general, positive emotions are more pronounced than negative ones, at least in terms of how they are reported.^1^^,^^27^ In addition, early studies of real-world emotions identified valence and arousal as the two dominant features of momentary affect consistently.^28^^,^^29^^,^^30^ We attempted to extend and improve on prior work by assessing the experiences of a larger and diverse sample of participants longitudinally using a more comprehensive set of scales and during a particularly turbulent time (the COVID pandemic and its associated events).

The studies thus far do not provide a consensus on the structure of subjective emotion experience.^31^^,^^32^^,^^33^ Importantly, the constraints of specific studies are likely to interact in producing divergent findings that have continued to fuel debates in the literature. For instance, discrete emotions have been reported to account for variance that is not captured by the standard valence and arousal scales—but on studies of response times to single words,^34^ which are a very different dependent measure in a very different task context than self-report of subjective feelings. Of course, all stimuli can be rated on discrete emotions, and such databases (including the ones we used for narratives^18^ and videos,^10^ as well as a number of others for single words in a range of different languages^35^^,^^36^^,^^37^^,^^38^^,^^39^) provide complementary characterizations of stimuli on discrete emotion labels beyond standard affective dimensions, such as valence and arousal. In fact, we included both discrete emotions and dimensional features in the present study and asked whether such emotion labels and the concepts they denote, when applied specifically to emotion experience as induced by potent stimuli, reveal a more continuous dimensional space or discrete clusters.

It is important to note that instances of emotion categories can be integrated into a dimensional framework when they are represented as points in a dimensional space.^40^ A dimensional approach does not at all preclude the discovery of categories; rather, it offers a quantitative framework to test for evidence of categories—are they objective categories represented as discrete clusters with firm boundaries or are they best understood as more conceptual or conventional categories that lack a data-driven basis^41^? We used precisely this approach in our study, quantifying emotion experiences in a dimensional space provided by our rating scales, and then testing whether the results showed any evidence of clusters that might constitute discrete categories.

To cast a broad net on the features that might characterize emotion experiences, we drew from a larger set of rating words from three main sources: standard emotion terms (e.g., the names for the so-called basic emotions, such as happiness and sadness),^9^ affective features (such as valence and self-relevance)^10^^,^^15^^,^^18^ as well as biologically inspired attributes (such as generalizability and persistence^42^). We were particularly interested to see whether the latter, biologically-inspired, features might reveal additional dimensions of variability. Importantly, we wanted to ensure that our rating terms captured as diverse a range of judgments as possible (while being non-redundant and clear in their meaning). To this end, we began with a broadly sampled set of around 70 terms and eventually reduced these to 28 rating scale labels which we verified to be representative of the initial set. We also verified that these 28 scales we used were in fact relatively high-dimensional in terms of their semantic meaning and thus did not artificially constrain the dimensionality of the emotion experiences that they were used to rate (see STAR Methods for details).

**Figure 1:**
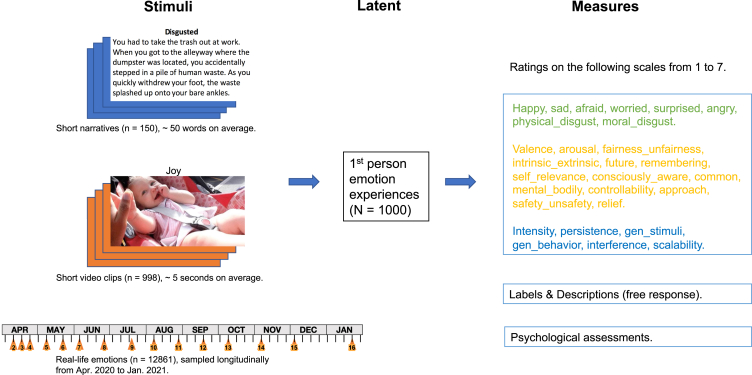
Overview of the study Emotions were evoked by three types of stimuli in a sample of 1,000 participants with comprehensive psychological assessments. Ratings were collected on scales inspired by different theories (green text: basic emotion words, yellow text: commonly used affective scales in the literature, and blue text: biologically-inspired features), as well as free text responses.

We used three types of emotion-eliciting stimuli: validated narratives^18^ and video clips^10^ from prior studies (sampled so as to maximize their diversity; see STAR Methods), as well as a rich array of real-life experiences sampled longitudinally during the COVID pandemic (Figure 1).^43^ We note that the three stimulus types differ in their degree of apparent selection bias. The narratives were constructed from scratch targeting 20 emotion categories and therefore can be seen as the most stereotypical stimuli for each pre-defined emotion category. The videos were curated from the internet with 34 emotion categories in mind, again leading to a set of relatively stereotypical examples. The real-world emotion, however, aimed to capture the full unbiased range of variation observed in everyday life. We do note here that, as with any study, our results are of course still limited both by the sampling of stimuli and of rating terms.

We started by quantifying the pairwise correlations between rating scales and asked whether the similarity structure was shared across the different types of inducing stimuli using representational similarity analysis (RSA). Given the correlations across rating scales, we further used exploratory factor analysis (EFA) to derive a small number of interpretable dimensions that capture most of the variance in the original high-dimensional space. We then probed the distribution of emotion experiences using dimensionality embedding and clustering techniques to visualize and identify clusters of emotions. Finally, enabled by the rich set of psychological background assessments in our participants, we provide a preliminary exploration of how individuals might differ in their emotion experiences evoked by our three stimulus types as a function of demographic and personality factors. Importantly, our aims to be as comprehensive as possible were motivated by a strongly data-driven approach. We did not set out to test any specific hypotheses or emotion theory, instead, we aimed to let the diversity of stimuli, ratings, and analyses speak for themselves.

## Results

### Similar representational structures across stimulus domains

**Figure 2:**
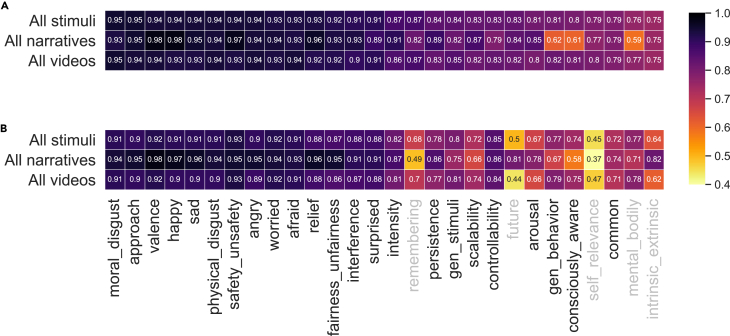
Evaluation of scale quality (A) Median test-retest reliability, and (B) median split-half reliability for 28 scales calculated using all evoked emotion experiment sessions, all narrative sessions, and all video sessions respectively. Scales were sorted based on median test-retest reliability across all stimuli. Five scales (in gray) were excluded because of low quality.

We assessed within-subject consistency (with Pearson correlations) and between-subject consensus (with split-half reliability) across scales (Figure 2, also see Figure S3 for full details). The pattern across scales was robust across experiment sessions and across evaluation metrics. Readability as evaluated using grade levels did not correlate significantly with scale quality as evaluated using median test-retest reliability (r = −0.19, *p* = 0.324) and median split-half reliability (r = −0.21, *p* = 0.295).

**Figure 3:**
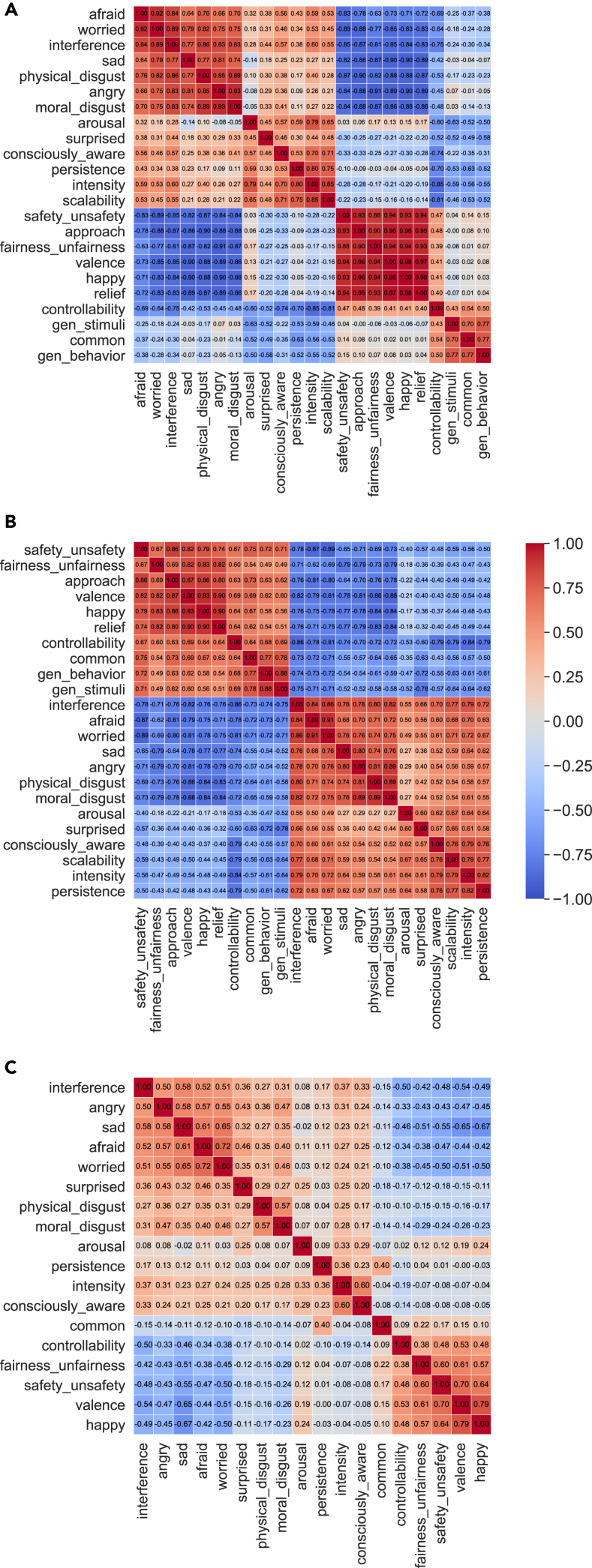
Representational structures across stimulus domains Correlation matrices for (A) emotions evoked by narratives (across 23 scales), (B) emotions evoked by videos (across 23 scales), and (C) real-life emotions (across 18 scales). Each matrix was sorted using hierarchical clustering to best visualize its underlying structure.

After excluding five scales due to their low reliability (see STAR Methods), we derived pairwise Pearson correlation matrices across the remaining scales for emotions evoked by narratives, emotions evoked by videos, and real-life emotions, respectively (Figure 3). We observed strong correlations across scales, suggesting that dimensionality could be reduced to represent the psychological space more efficiently. Most consistently, across all of the stimulus domains, two correlated groups of scales emerged: those scales whose higher ratings indicate emotions to be more negatively valenced (*afraid*, *worried*, *physical disgust*, *angry*, and *moral disgust*) and those scales whose higher ratings indicate emotions to be more positively valenced (*valence*, *happy*, *safety*, and *fairness*). Note that most scales were either not committed to any particular valence or bivalent, thus capturing both negative and positive valence.

Figure 3 further suggested that emotion experiences across stimulus types shared a similar broad correlation structure. To make a formal comparison across all three stimulus types, we used only those 18 rating scales that were shared across all three stimulus domains, confirming the visual impression that the representational structure was highly consistent across stimulus types (Figure S4; second-order rs = 0.953, 0.923 and 0.909 for narratives and videos, narratives and real-life, and videos and real-life, respectively (all ps < 0.0001)).

### Low-dimensional spaces underlie emotion experience

To represent the psychological space using a smaller number of underlying factors, we performed exploratory factor analysis. The number of factors (dimensions) to retain is well known to be indeterminate, and a number of metrics are commonly used in the literature: the Very Simple Structure, empirical BIC, Velicer’s MAP, parallel analysis, the acceleration factor, and the optimal coordinate metric, all of which we used. However, these criteria do not always agree since they are based on different assumptions, and we therefore prioritized an entirely data-driven approach: empirical cross-validation where we applied EFA to half of the data and confirmatory factor analysis (CFA) to the other half. To ensure the robustness of our results, we also systematically decimated both the number of stimuli and the number of scales, testing for the stability of the results if the analysis was re-done on a randomly sampled subset of the data. Finally, we considered the interpretability of the final results (see STAR Methods for complete description of our analyses).

For emotions evoked by narratives, results from the six commonly used statistical tests suggested either 2 or 3 factors to retain. The data-driven cross-validation procedure (Figure S5A) showed that two factors were most appropriate: there was a significant improvement in explained variance from EFA as well as model fit from CFA as the number of factors increased from 1 to 2, but adding further factors subsequently showed only marginal improvement. We next assessed the robustness of the 2 factor and 3 factor solutions and found both to be robust with regard to the number of stimuli and number of scales (Figure S6A). We thus decided to retain 3 factors for completeness.

For emotions evoked by videos, results from the six commonly used statistical tests again did not converge to a single number but suggested 1 or 4 or 6 factors to retain. The cross-validation procedure suggested that 2 to 4 factors all seemed reasonable (Figure S5B). We further assessed the robustness of the 3 factor and 4 factor solutions (Figure S6B). Both were robust with regard to the number of stimuli, but the 4 factor solution was not robust with regard to the number of scales (the “safety” factor was unstable). We thus decided to retain 3 factors.

For real-life emotions, results from the six commonly used statistical tests did not converge to a single number but suggested 1 or 2 or 6 or 8 factors to retain. The cross-validation procedure suggested that 2 to 5 factors all seemed reasonable (Figure S5C). We further assessed the robustness of the 3 factor and 4 factor solutions (Figure S6C). Both were robust with regard to the number of stimuli, but the 3 factor solution was not robust with regard to the number of scales (the “negative affect” factor was unstable). We thus decided to retain 4 factors.

**Figure 4:**
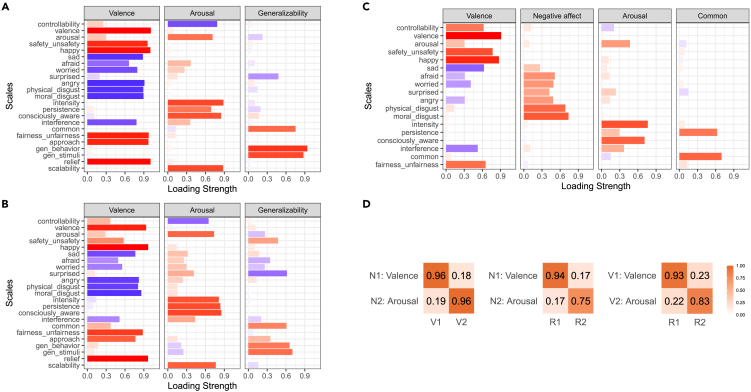
Dimensionality of emotion experiences across stimulus domains Factor loadings for (A) emotions evoked by narratives, (B) emotions evoked by videos, and (C) real-life emotions. Each column plots the strength of the factor loadings (x axis, absolute value) across scales (y axis). Color indicates the sign of the loading (red for positive and blue for negative); more saturated colors for higher absolute values. (D) Tucker indices of factor congruence (with orthogonal Procrustes rotation) across stimulus types (N: narrative, V: video, and R: real-life). The first factor (N1/V1/R1) is valence, and the second factor (N2/V2/R2) is arousal.

We then used EFA to extract three, three, and four factors for the narrative-evoked, video-evoked, and real-life emotions, respectively, using the minimal residual method; solutions were rotated with oblimin for interpretability (see Figure 4 for factor loadings). For emotions evoked by narratives, the three factors, interpreted as “valence”, “arousal”, and “generalizability”, each explained 48%, 21%, and 13% of the common variance in the data (82% in total, 84% in total if four factors were extracted). For emotions evoked by videos, the three factors, interpreted again as “valence”, “arousal”, and “generalizability”, each explained 41%, 24%, and 15% of the common variance in the data (81% in total, 83% in total if four factors were extracted). Finally, for real-life emotions, the four factors, interpreted as “valence”, “negative affect”, “arousal”, and “common”, each explained 25%, 13%, 10%, and 5% of the common variance in the data (54% in total, 58% in total if five factors were extracted). These quantitative results demonstrate the validity of a low number of about 3 dimensions in our data, as going to additional dimensions provides only incremental improvements in explained variance.

Note that the “valence” and “arousal” factors were identified across all stimulus domains, in line with previous literature.^6^ The “generalizability” factor is novel to the best of our knowledge. The “common” factor describes how common and persistent emotions are and is probably related to the “generalizability” factor, which we did not properly assess in real-life emotions (the specific scales with highest loadings on the generalizability factor were not included for assessing real-life emotions as we deemed them to be inapplicable).

A specific set of scales (*disgust*, *fear*, *surprise*, and *anger*) loaded strongly onto the “negative affect” factor that was uniquely seen for the real-life emotions. One interpretation is that this factor was specific to experiences during the COVID pandemic (and all the other stressors associated with it) during which we sampled real-life emotions. The temporal pattern of the factor across the longitudinal waves of data collection (Figure S7) revealed a generally decreasing trend (in contrast to the severity of the pandemic measured by deaths and cases) which may indicate that people adapted to the pandemic over time as restrictions relaxed and mask use increased. In addition, there was a notable peak around wave 7 which was the closest data collection we had (June 6 and 7, 2020) to the incident of George Floyd’s death. We observed differences on this factor with respect to gender, geographic region, and political party affiliation. Females, people residing in the West and Northeast, and Democrats scored higher than males, those in the Midwest and South, and Republicans (Figure S8).

Noting the consistency of the overall correlation structure and the semantic similarity of the factors across stimulus types, we directly tested the idea of shared latent factors across stimulus domains (see Figure S9 for the determination of the number of factors that would best explain the shared structure). Using the correlation matrices across 18 shared scales, we extracted two factors from each of the three types of data and quantified the relatedness of the factors by calculating factor congruence (Figure 4D). A two-dimensional structure of emotion experience was indeed consistent across stimulus types as indicated by high levels of factor congruence.

To address possible concerns that our results regarding the number and nature of affective dimensions may depend on the use of EFA, we further analyzed our data using several dimensionality reduction techniques commonly used in prior work (see STAR Methods for details).^10^^,^^44^^,^^45^ All methods (principal components analysis (PCA), autoencoders with cross-validation, and principal preserved component analysis (PPCA)) suggested a small number of dimensions to keep on the basis of cumulative proportion of variance explained (Figure S16). We further note that the nature of the first few dimensions was highly consistent across these different analytic methods.

When extracting the same number of components using PCA, we largely reproduced the same factors that we previously extracted using EFA (Tucker indices of factor congruence: 0.99, 0.96, and 0.96 for emotions evoked by narratives; 0.96, 0.96, and 0.96 for emotions evoked by videos; 0.98, 0.97, 0.98, and 1.00 for real-life emotions). The dimensional representation learned by the autoencoder model (with linear-linear activation functions) also reproduced the dimensions from EFA (Mean Tucker indices of factor congruence between the factor loadings from EFA and the autoencoder’s decoder layer weights: 0.99, 0.97, and 0.96 [SDs = 0.01, 0.03, and 0.04] for emotions evoked by narratives; 0.99, 0.97, and 0.96 [SDs = 0.01, 0.02, and 0.01] for emotions evoked by videos; 0.98, 0.96, 0.96, and 0.98 [SDs = 0.01, 0.02, 0.03, and 0.01] for real-life emotions). Lastly, PPCA suggested 14 and 16 dimensions to be significant (using one tailed Wilcoxon signed-rank test, Bonferroni-corrected α = 0.00217) for emotions evoked by narratives and videos respectively (as repeated measures for each rating was required, this analysis was not applicable to real-life emotions). Though suggesting very different total numbers of dimensions to retain based on a metric of statistical significance, the first several components from the PPCA remained remarkably similar to the factors from EFA (Pearson’s correlations between unrotated factor scores from EFA and unrotated component scores from PPCA: 0.99, −0.97, and 0.89 for emotions evoked by narratives; −1.00, −0.98, and 0.99 for emotions evoked by videos). In the aggregate, these several different analyses provide the most convergent result that emotion experiences are characterized efficiently by a low-dimensional space that includes valence and arousal.

### Continuous gradients of emotion experience

To examine the distribution of emotion experiences in the psychological space, we first projected data from the original high-dimensional spaces to two-dimensional spaces for easier visualization and interpretation. We further combined two sources of information (the categorical labels for the intended emotion categories and the factor scores) with Uniform Manifold Approximation and Projection (UMAP) plots to address the key question of whether emotion experiences are discrete or dimensional.

**Figure 5:**
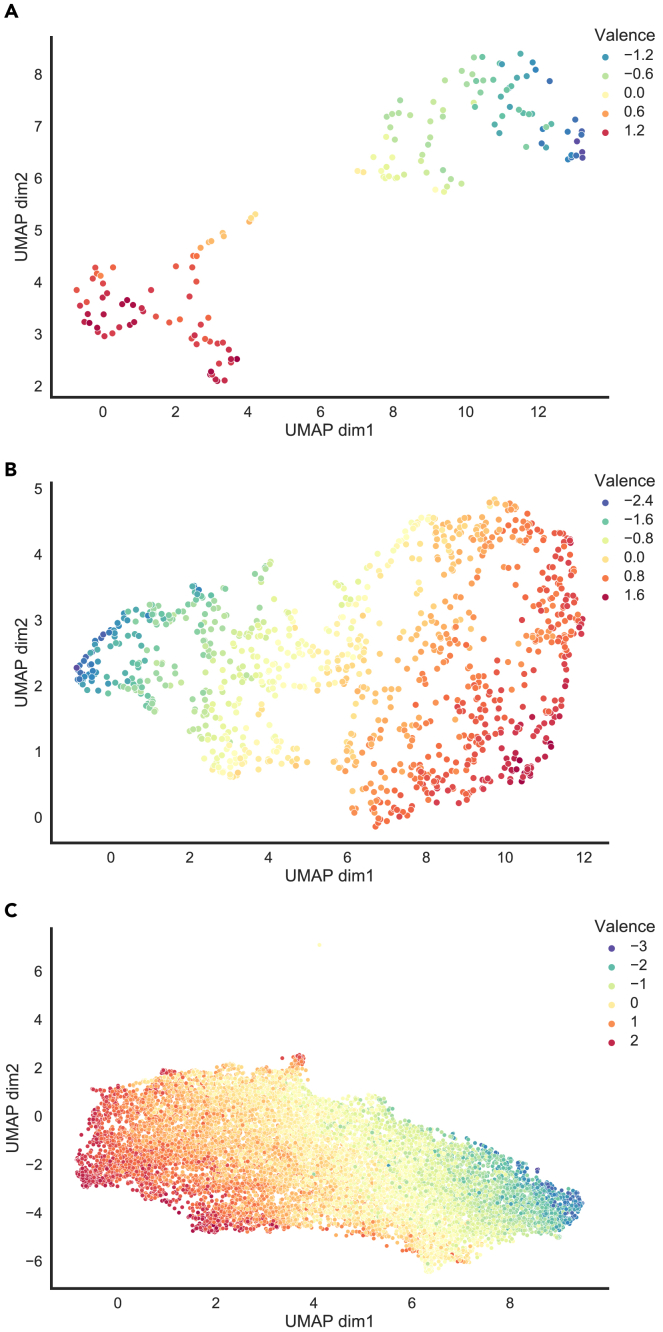
Distribution of emotion experiences across stimulus domains Uniform Manifold Approximation and Projection (UMAP) plots for (A) emotions evoked by narratives, (B) emotions evoked by videos, and (C) real-life emotions. Each dot represents one emotion experience, color-coded for the “valence” factor as indicated.

Across all three types of emotion experiences, valence emerged as a continuous global gradient (Figure 5), in line with the dimensional view of emotions. Emotions varied along other dimensions smoothly as well but not in a single direction as for valence (Figures S10–S12). Visualizations revealed that extremely positive or negative emotions are more intense and arousing compared to neutral ones.

For emotions evoked by narratives and videos, the UMAP plots were also visualized with the categorical labels from the original studies from which we obtained the stimuli in the first place (Figures S10 and S11). We observed that for some emotions, instances belonging to the same categories were located in closer proximity to one another compared to other categories where instances were more scattered.

Note that in addition to rating on the scales, participants were also asked to provide emotion labels as free text responses (see STAR Methods for details). So that readers can explore these results in more detail for their own interpretation, we also provide interactive versions of UMAP plots where each emotion experience can be inspected with the free responses collected in our study when users hover the mouse over the plots (narrative: https://ellenhan0201.github.io/project/emotion/narrative.html; video: https://ellenhan0201.github.io/project/emotion/video.html; real-life: https://ellenhan0201.github.io/project/emotion/reallife.html).

### No discrete clusters of emotion experience

UMAP visualizations, especially the global gradient of valence across all stimulus domains, supported a dimensional rather than discrete categorical view of emotions. Nonetheless, as loss of information is inevitable for any dimensionality reduction tool, it remains possible that well-separated clusters in the high-dimensional space overlap in the low-dimensional embeddings. The qualitative observation of no discrete clustering structure from UMAP was supported by inspecting the distributions of pairwise Euclidean distances in the original high dimensional spaces (Figure S18). Additionally, Hartigan’s dip test for unimodality (see STAR Methods) suggested unimodal structure for emotions evoked by videos and real-life emotions (dip statistic = 0.001, *p* = 0.992 for emotions evoked by videos; dip statistic = 0.001, *p* = 0.995 for real-life emotions). We note that for emotions evoked by narratives, there was a bimodal structure that is likely explained by the fact that these narratives were specifically designed to evoke a roughly balanced number of positive and negative emotions (dip statistic = 0.007, *p* = 0 for emotions evoked by narratives). In no case did we observe numerous peaks in the distributions that might have corresponded to discrete emotions.

**Figure 6:**
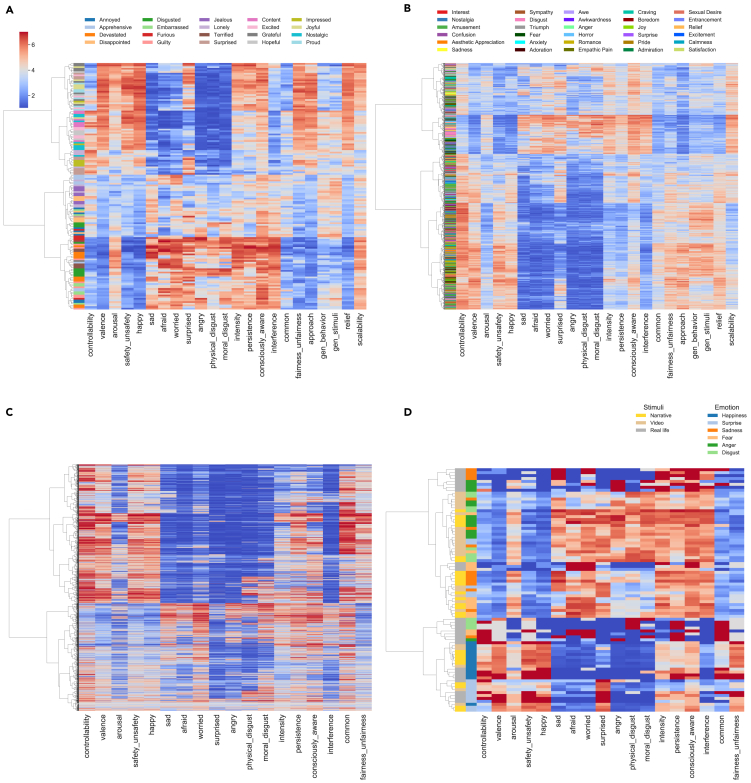
Hierarchical structure of emotion experiences Hierarchical clustering with ratings (columns except those otherwise noted) for each emotion experience (each row) for (A) emotions evoked by narratives (first column indicates the intended emotion category), (B) emotions evoked by videos (first column indicates the dominant emotion category), (C) real-life emotions, and (D) basic emotions (first column indicates the stimulus type and second column indicates the emotion category).

To address whether the categories as defined semantically actually form clusters in the high dimensional space with clear boundaries more directly, we probed an extensive hierarchy of clusters, using hierarchical agglomerative clustering (Figures 6A–6C). We utilized the ratings and the free descriptions of the emotion labels to further interpret the meaning of the clusters. In principle, the hierarchy of clusters can be probed at all possible levels, with the bottom level having a single instance as its own cluster. However, it should be noted that each solution represents a different level of fit and having more clusters does not necessarily imply better interpretation.

For emotions across all three stimulus domains, the basis of partitioning at the top of the hierarchy was valence, resulting in two clusters of positive and negative emotions respectively. For emotions evoked by narratives, the second level of partitioning was based on arousal, resulting in four clusters: strong positive emotions, weak positive emotions, weak negative emotions, and strong negative emotions from top to bottom, respectively. For emotions evoked by videos, positive emotions were further partitioned on the basis of generalizability while negative emotions were further partitioned on the basis of arousal. Cluster solutions further down the dendrogram were harder to interpret. Inspecting the row labels that encode the intended categories, we observed both within-category similarity (as indicated by some sequential rows of the same color) and cross-category similarity (as indicated by a mixing of colors) for stimulus-evoked emotions.

A second approach to quantifying whether there were discrete clusters of emotion experience is not data-driven but uses the very same discrete emotion labels as those in the original studies from which we derived our stimuli (narratives and videos). We thus next asked whether we can recover the intended emotion categories from the original studies (20 categories for emotions evoked by narratives and 30 categories for emotions evoked by videos) as clusters using the K-means clustering algorithm. The solutions extracted by K-means were in low agreement with the intended categories (for emotions evoked by narratives: adjusted rand score: 0.244, adjusted mutual info score: 0.395, see Figure S13A for the contingency matrix; for emotions evoked by videos: adjusted rand score: 0.08, adjusted mutual info score: 0.254, see Figure S13B for the contingency matrix).

We next restricted ourselves to instances of emotion experiences clearly belonging to one of the six basic emotion categories (see STAR Methods for selection criteria) and performed hierarchical clustering on this specific set of emotion experiences (Figure 6D, also see Figure S14 for clustering on averaged basic emotions). First, we observed no prominent clustering based on stimulus type, though similarities within the same stimulus type were indicated by some blocks of rows of the same color. In addition, we observed that even for these instances that were specifically chosen as the best examples of basic emotions, the intended basic emotion categories were not perfectly recovered. Among the six basic emotions, happiness and surprise clustered relatively better than the negative emotions.

### Individual differences in emotion experiences

**Figure 7:**
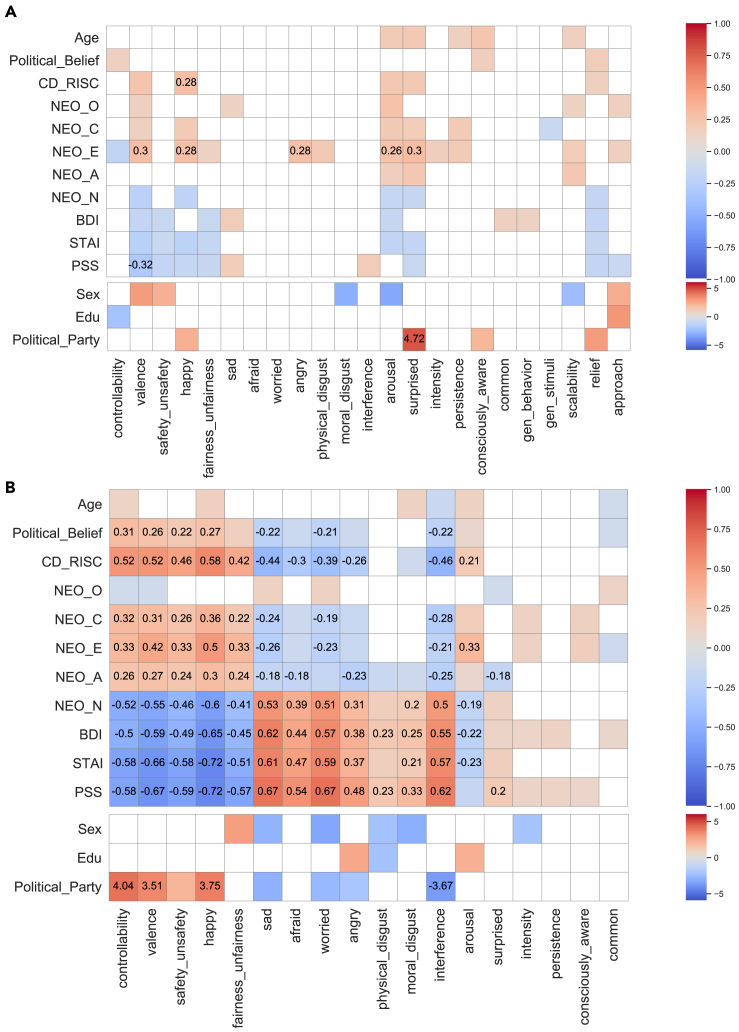
Individual differences in emotion experiences Pairwise Pearson’s correlations (upper) between ratings and demographic and psychological variables, and Welch’s t test (lower) for means of ratings of different groups (divided based on sex, education and political party; t-statistics for males - females, high - low education, and republicans - democrats respectively) for (A) stimulus-evoked emotions (approximately 180 participants for each individual test, depending on the data available for each scale after exclusion), and (B) real-life emotions (441 participants for each individual test). Raw results with *p* < 0.05 are colored (otherwise masked) and Bonferroni corrected results with *p* < 0.05 are annotated with value.

An important topic for future studies will be to investigate individual differences among the participants, especially cultural and language differences. This was not an aim of the present study, but we nonetheless collected a variety of other measures from our participants, which we briefly summarize here as exploratory findings that could motivate future studies focused on individual differences. We examined whether there were meaningful individual differences in terms of the mean magnitude of ratings. The overall pattern indicated that there were a few variables showing individual differences for real-life emotions but not for emotions evoked by narratives or videos (Figure 7, see also Figure S15 when we corrected real-life emotion ratings with potential baseline rating bias from narrative/video ratings). The most notable exception in terms of stimulus-evoked emotions was that extraverted individuals tended to experience more intense emotions regardless of valence.

For real-life emotions, individuals who were more politically conservative, resilient, conscientious, extraverted, or agreeable experienced more positive emotions. On the other hand, individuals who were more neurotic, depressed, anxious, or stressed experienced more negative emotions. These patterns for the real-life emotions are perhaps unexpected and might be attributable either to individual differences in the real-world environment in which people find themselves (e.g., resilient and extraverted people might tend to end up in situations mostly evoking positively valenced experiences, whereas neurotic and depressed people end up in situations that evoke negatively valenced experiences), and/or in the actual experiences and their conceptualization, irrespective of the inducing situations (e.g., neurotic and depressed individuals see the world more negatively in general). Contrary to some prior studies,^46^ we did not find a significant effect of age, possibly due to the restricted age range of our participants (Table S2). Notably, our sample included few individuals over 65, an age group reported to show a marked increase in valence ratings.^46^ Future studies will also need to compare our present findings of individual differences with those reported on performance tasks (such as effects on processing speed as evident in eye movements while reading emotional sentences or narratives^47^^,^^48^ or reaction times and accuracy rates for emotional word recognition in a lexical decision task^49^).

## Discussion

Using a rich set of scales, we characterized the psychological space of emotion experiences evoked by a validated set of narratives, a validated set of videos, and actual experiences in real life during 2020. All three types of emotion experiences shared similar underlying low-dimensional structure (Figures 4 and S9), with the first two factors accounting for most of the variance corresponding to valence and arousal, in line with prior work.^6^ Factors related to generalizability also emerged, but the additional proportion of variance accounted for by higher-order factors was minimal, demonstrating that a psychological space of 2–3 dimensions explained most of the variance in our data. Characterizing the distribution revealed that emotions were distributed along continuous gradients (Figures 5 and S10–S12), with no well-separated clusters even for emotions belonging to the six basic emotion categories (Figure 6), contrary to theories postulating discrete emotion categories.^50^ In addition, individual differences were explored, and we found associations with psychological and demographic variables for real-life emotion experiences but not for emotion experiences evoked by narratives or videos (Figure 7).

We stress that the aforementioned findings were obtained through a comprehensive set of analyses that converged on a consistent conclusion. We used data-driven cross-validation to determine the number of factors to retain (Figure S5), compared this with six commonly used metrics, checked the robustness of the results with respect to data quantity by sub-sampling both stimuli and rating scales to ensure that the factor solutions were stable (Figure S6), and supplemented our factor analysis with additional dimensionality reduction methods such as PCA and autoencoders (Figure S16). Finally, we verified that the specific rating scales we used did not artificially limit the number of dimensions we could have obtained, through an analysis of their semantic similarity (Figure S17). This comprehensive approach, together with our large participant sample and our starting aim of using three very different emotion inductions (narratives, videos, and real life), argues in favor of the robustness and generalizability of our findings.

So far, we have highlighted the remarkable level of similarity of emotion experiences across different domains of stimuli as indicated by highly consistent representational structures (Figures 3 and S4), shared factors (valence and arousal) accounting for the majority of variance (Figure 4), and a shared pattern of distribution along a continuous global gradient of valence (Figure 5). However, a closer look revealed subtle differences across domains as well. Despite the similar overall structure, the magnitudes of the correlation coefficients in the correlation matrices did differ, with the strongest overall correlations for emotions evoked by videos and the weakest correlations for real-life emotions (Figure 3). The strong correlations of videos likely arise from the fact that videos are diverse and potent and thus evoked intense emotions, helping to sharpen the correlation structure. On the other hand, possible explanations for real-life emotions having the weakest correlations could be that data were simply noisier as aggregation across participants was not possible (as every individual’s experience was idiosyncratic) and real-life emotions were typically weaker and less intense than what can be depicted in video.

Averaging the different emotion experiences of each basic emotion category for each stimulus type and then clustering on these averaged basic emotions allowed us to better examine the effect of stimulus domain (Figure S14). In general, emotions evoked by narratives and videos were more similar to one another than to those experienced in real life. The differences between stimulus-evoked emotions and real-life emotions were most prominent for the negative emotions, and less so for happiness and surprise. The separate cluster at the bottom revealed that the negative emotions (sadness, anger, fear, and disgust) in real life were less surprising, less arousing, more controlled, more common, and also purer than the ones evoked by narratives or videos. These distinctions between real-life emotions and the stimulus-evoked emotions might be explained by a better understanding of one’s own emotions when they occur in a more complete context.

Our study discovered a dimension not previously reported, which based on its loading onto our ratings scales we have interpreted as “generalizability”, arising from the biologically inspired ratings we had included^42^—*generalizability over stimuli*, *generalizability over behavior*, as well as the scales *common*, and *surprised*. This dimension is less readily interpretable than valence and arousal. We would speculate that it might be related to the degree to which an emotion is relatively modular and domain specific or domain general. We find it intriguing that Herbert Simon, in his famous paper articulating an engineering perspective on emotions,^51^ speculated a similar functional role for emotions: they should be able to quickly interrupt ongoing processing across a range of stimuli and behaviors, some of which would be expected to be tightly domain-specific (such as seeing a snake or tiger) and some more open-ended (Simon speculated that social contexts might be particularly relevant here). Low generalizability would characterize those emotions that can only occur in specific situations; high generalizability would characterize those emotions triggered by many different situations and thus be more common.

The shared dimensions produced across our three inducing stimuli (narratives, videos, and real life) pose the question of how such a common structure might arise. At the level of sensory input, the stimuli were very different, and diverse even within a stimulus category. At the level of rating scale output, our rating scales were also diverse and relatively high dimensional (Figure S17). We suggest that the common low dimensionality arises between sensory processing and conceptualization/verbalization: at the level of conscious experience of the emotion. On this view, our emotion experience provides a common workspace where diverse stimuli can generate diverse ratings, but the experience itself is relatively low dimensional. Of course, we can be aware of many other aspects of the stimuli, but these would not be part of the emotion experience as such. Our interpretation is consonant with views in the psychology of emotion that describe emotion experience in terms of “core affect”: an underlying dimensional space for emotion experience that is low dimensional (typically two-dimensional in most theories^5^^,^^6^^,^^7^). On the other hand, we note that some studies of emotion concepts (as derived from words as the stimuli) also find surprisingly low-dimensional results.^52^

This interpretation has important implications for taxonomies of emotion, on which there is vigorous debate. As we reviewed in our Introduction, there are debates both about whether emotions are discrete (categorical)^8^ or continuous (dimensional),^5^ and about the number of categories or dimensions.^10^ Our approach in the present study was data-driven, and we consequently did not have any specific view or theory as a prior hypothesis to test. Instead, we attempted to be as broad and inclusive as feasible, surveying 3 very different stimulus types, on a highly diverse set of rating scales, using a series of different methods (EFA, PCA, autoencoders, PPCA), with explicit tests both for the possibility of clusters that might emerge in a data-driven fashion as well as tests for the specific emotion categories designed by the prior studies from which we obtained our stimuli (the narratives and videos). The upshot from our data-driven approach is that we find no evidence for discrete emotion clusters and that instead emotion experience appears to be well described by a low-dimensional continuous space.

To reconcile this conclusion with contrary views, we note again that our approach was atheoretical—a specific emotion theory might very well place constraints on the design, analysis, and interpretation of our study, so as to obtain different conclusions. Many (perhaps most) prior studies have relied on a relatively small number of prototypical (or stereotypical) examples and have interpreted classification results statistically above chance as strong evidence for discrete emotions.^21^^,^^22^^,^^23^^,^^24^ This approach can be misleading, as the preconceived emotion categories designed into the experiment by the investigators may shape the dimensionality they eventually identified. It has also been argued that attributing the considerable level of within-category variability and cross-category similarity solely to noise seems problematic.^53^ Our study differs from these previous studies by not restricting ourselves to prototypical examples that maximally differentiate emotion categories, especially our real-life emotions. Notably, in our data, even the selected best examples of basic emotion categories did not form distinct clusters, especially for negative emotions (Figure S6D). This observation suggests that the boundaries between emotion categories are not sharp (although they can be fuzzy), a point acknowledged even among those who endorse a discrete emotion perspective.^10^^,^^54^ To summarize, while we acknowledge that taxonomies are crucial for a nuanced understanding of emotions, the question of the nature of emotion categories (that is, how “real” they are in a data-driven sense) remains open. We hope that the comparative comprehensiveness of our study (a large number of stimuli drawn from three different categories, a large sample size of participants, and several different analyses, that all converged on the same conclusions) helps to inform ongoing debates.

To conclude with our interpretation of the findings: there is no question that we have a very large number of words and concepts with which to describe and think about emotions; these vary across cultures and languages^55^^,^^56^ and may show further conceptual structure, such as distinctions between emotions and sentiments.^57^ That was not the topic of our study. There is also no question about the richness of effects that emotions have on cognition and behavior (such as the effects of emotion on decision-making,^58^ on attention^59^^,^^60^ and on memory^59^^,^^61^); also see the literature on cognition-emotion interactions.^62^ That was also not the topic of our study. Our study was very explicitly about subjective experience of induced emotions, as opposed to the semantics of emotion concepts, or the functional ways in which emotion can interact with cognition. While we can think about and write about emotions using a very rich palette of concepts and words, and while emotion-elicited behavior is extremely varied and context dependent, we found that the subjective experience of emotion was best characterized by a continuous and low-dimensional space. A possible analogy may be found in color perception: we have a large number of color words (again, depending on language and culture^63^) but the underlying psychological color space is in fact continuous and low dimensional (in that example, imposed by the number of spectrally distinct photoreceptors we have in our retina). We once again note that our conclusions were data-driven and thus ignore possible constraints that might be imposed by particular emotion theories, and furthermore acknowledge that our approach features a number of important limitations that we detail next.

### Limitations of the study

There are several important limitations to note. First, the current study is a psychological investigation of people’s emotion experiences, more specifically, people’s judgments about their conscious experiences of emotions, focused entirely on self-report. It is important to note that a range of performance-based measures has also been used in the emotion literature, such as the processing speed of emotional words using a lexical decision task.^34^^,^^49^ It would be interesting to compare the present findings using self-report questionnaires with such performance task measures, since these two types of dependent measures have been reported to cluster as relatively unrelated.^64^

Second, although we aimed for a comprehensive sampling of rating scales (whose semantic similarity was examined to make sure they were diverse), this is of course not a complete list. Theoretically, it is possible that one might identify more dimensions if more scales were included (although this becomes complicated since one would need to articulate some theory to decide which words describe emotions and which might describe aspects of conscious experience that are not considered constitutive of an emotion^55^^,^^65^^,^^66^). We do note that we included both positively and negatively valenced attributes in our ratings—several scales were bivalent on valence (e.g., *approach*, *safety*, *relief*, and *fairness*), and most of the other scales are agnostic with respect to valence (e.g., *intensity*, *arousal*, *persistence*, etc. do not have any apparent valence bias). Nonetheless, it would be important in future studies to probe especially positively valenced scales in more detail, given substantial findings about variety in this domain that is typically undersampled in most studies (see Shiota M.N.^54^ for review).

Third, there are also limitations inherent in the stimuli we used. For the narratives that we used whose intended emotion can be recognized, participants may simply rate what the intended emotion is supposed to be rather than reflecting on their conscious affective experiences. Conversely, the videos that we used are able to elicit strongly felt emotions, but participants may not want to have those experiences and may thus downregulate their actually experienced emotions. In both cases, what it is that participants report on may be more related to what it is that they think one should feel, or what they would like to feel, than what they would actually feel.

A fourth limitation inherent to all studies that use dimensionality reduction methods such as factor analysis is that the dimensions produced are indeterminate. There is an inherent trade-off between the number of dimensions retained and the variance explained: preserving a greater number of dimensions would of course account for a higher proportion of variance, but one must also consider the efficiency of representation. We used a range of methods (including standard statistical tests based primarily on the scree plot, a completely data-driven cross-validation approach, and thorough assessment of the robustness of the factor solutions) to provide a comprehensive picture. Nonetheless, the low number of factors we obtained, and their interpretation, are limited by the stimuli and rating scales used in the first place and might be judged differently even given our data by somebody with a strong *a priori* theoretical constraints (we had none).

Finally, the data of our study were collected solely from an English-speaking sample of adult participants residing in the US. We acknowledge that cultures and languages may influence the structure of emotion experiences, as may other individual differences, a topic we only explored in a very cursory fashion in the present study. It will be important for future studies to keep all the aforementioned limitations in mind as we build a cumulative affective science that can generalize over stimuli, methods, and participants.

## STAR★Methods

### Key resources table

**Table:** 

REAGENT or RESOURCE	SOURCE	IDENTIFIER
**Deposited data**		

Video Stimuli	Keltner Lab	https://goo.gl/forms/XErJw9sBeyuOyp5Q2
Narrative Stimuli	adapted from Saxe Lab	https://osf.io/u83b4
Experimental data	This paper	https://osf.io/azxyk/

**Software and algorithms**		

Analysis scripts	This paper	https://osf.io/mwp36/

### Resource availability

#### Lead contact

Further information and requests for resources and reagents should be directed to and will be fulfilled by the lead contact, Yanting Han (yhhan@caltech.edu).

#### Materials availability

This study did not generate new unique reagents.

#### Data and code availability

• All de-identified data have been deposited on the Open Science Framework at https://osf.io/azxyk/. They are publicly available as of the date of publication. The videos and narratives used in this study are adapted from previous studies, and can be accessed at https://goo.gl/forms/XErJw9sBeyuOyp5Q2 and https://osf.io/u83b4 respectively.
• All original code has been deposited on the Open Science Framework at https://osf.io/mwp36/and is publicly available as of the date of publication.
• Any additional information required to reanalyze the data reported in this paper is available from the lead contact upon request.

### Experimental model and study participant details

#### Participants

The same participant pool was utilized to study emotions evoked by narratives and videos, and real-life emotions (as part of the COVID-Dynamic study: https://coviddynamic.caltech.edu). All studies in this report were approved by the Institutional Review Board of the California Institute of Technology and informed consent was obtained from all participants (Protocol Number: IR19-0932). The COVID-Dynamic study was pre-registered before data collection began (at https://osf.io/sb6qx), and details about the dataset can be found in the data release paper.^43^ Briefly, the recruitment was done through Prolific (www.prolific.co) and participants were required to be adults 18 or older, fluent in English, and reside in the United States. In addition, they had to have a Prolific approval rating of 98% or higher, and a minimum of 5 Prolific studies completed. In total, 1797 participants completed Wave 1 of the COVID-Dynamic study. The study of real-life emotions was conducted with all COVID-Dynamic participants during waves 2–16.

All COVID-Dynamic participants were invited to enroll in the narrative and video rating experiments (which were conducted independently from COVID-Dynamic waves). We predetermined the sample size to be 15 participants per scale prior to the main data collection (see registration at https://osf.io/vprz8). We estimated our sample size based on a recent study about the point of stability for impression formation from faces^67^ which introduced a sampling procedure to determine when the average of observations would be stable. Given that the rating scales ranged from 1 to 7, the corridor of stability (COS) deemed acceptable was +/− 0.5 and the level of confidence deemed acceptable was 80%. Using pilot data which involved three scales of different semantic complexity, we found that 15 participants would be enough for even the most complicated scale to satisfy the above criteria. Expecting attrition from data quality exclusions, we increased the sample size to 20 participants per scale. Each of the 12 sessions (2 narrative, 10 video) used a fixed stimulus set, which was rated on 28 scales by 20 participants per scale (i.e., 560 participants per study). All COVID-Dynamic participants were invited and were enrolled in our study in the order in which they applied.

#### Exclusion

##### Evoked emotion experiments

We pre-registered the exclusion criteria for the evoked emotion experiments (using narratives and videos) before data collection began (https://osf.io/vprz8). The exclusions were applied at multiple levels (see counts after each level of exclusion in Figure S1A).

Trials were excluded if responses were timed-out or if reaction time was extremely short (<400 ms). Sessions were excluded if any of the following conditions was met: failing more than 1 attention check (out of a total of 3 checks); extremely low test-retest reliability estimated from the retest session (below 3 standard deviations from the mean reliability compared to all participants who rated the same scale on the same set of stimuli) or having more than 10% of invalid trials. Participants were excluded if they had more than 3 invalid sessions out of all the sessions that they did.

We added further exclusions at the participant level from the exclusionary criteria of the real-life emotion sampling experiments (which we describe below) because of the shared participant pool, which was not planned at the time of pre-registration.

##### Emotions sampled in everyday life

The exclusion criteria were chosen based on pilot analysis conducted using data from 50 randomly selected participants (from a total sample of 1797 participants) across 16 waves of data collections and pre-registered (https://osf.io/y78mz) before applying to the entire dataset. Again, exclusions were applied at multiple levels (see counts after each level of exclusion in Figure S1B).

Wave-wise exclusions were based on the data quality metrics (for detailed info, please see the data quality section in ref.^43^). Specifically, data from a wave was excluded if a participant failed 2 or more attention questions or the percentage of quality checks failed was equal or higher than 20%.

Participant-wise exclusions were based on multiple criteria to ensure data quality. First, participants were excluded if they self-reported that they were diagnosed with any of the following mental health conditions not related to our hypothesis: schizophrenia, bipolar disorder, or posttraumatic stress disorder; or multiple comorbid psychiatric conditions other than depression and anxiety. Second, participants were also excluded if they had 3 or more waves where their data were deemed as low quality. Third, participants were excluded if they had completed less than 50% of all waves (8 waves). And lastly, given that the real-life emotion sampling experiments and the evoked emotion experiments shared the same participant pool, a participant was excluded in both datasets if they met the exclusion criteria from either of the studies.

The number of participants after exclusion for the narrative rating experiments, the video rating experiments and the real-life emotion sampling experiments were 554, 638 and 1000 respectively (note that these participant sets overlap with one another). Among the 1000 participants, there were 493 males, 507 females; mean age = 39.65 years, SD = 14.22 years; 0.2% American Indian/Alaska Native, 10.5% Asian, 0.1% Native Hawaiian or Other Pacific Islander, 7.1% Black or African American, 74.9% White, 5.1% Multiracial, 1.4% Other, 0.7% Prefer not to disclose, see Table S2 for further details.

### Method details

#### Scale selection

Our goal was to have a maximally diverse (but non-redundant) set of scales that describe the properties of emotion experiences. We started by assembling an inclusive list from multiple sources: the words for basic emotions,^9^ affective scales from modern studies,^10^^,^^15^^,^^18^ and biologically-inspired features.^42^ Notably, we included several scales that are based on a biological consideration of the functional properties of emotions, such as their need to persist over time, take over volitional behavior, and generalize across stimuli and behaviors (all criteria that distinguish emotions from mere reflexes). These scales derive from prior work comparing emotions across species, notably the book, “The Neuroscience of Emotion: A New Synthesis”.^42^ Our initial broad sampling from these references initially produced 70 terms. We next excluded those scales that described context rather than emotion, and combined synonymous and antonymous scales to eliminate redundancy. This resulted in a set of 28 scales, for which we generated clear definitions to provide unambiguous interpretation. The definitions of the scales along with the readability of the scales are included in the supplementary materials (Table S1). Readability was calculated using the Flesch–Kincaid grade levels,^68^ a test based on word length and sentence length. Most of the scales required a grade level of 12 and lower, which corresponds to a high school education.

We derived sentence embedding for our 28 scales using the Universal Sentence Encoder^69^ of the definitions in Table S1 (basic English stop-words and words that were present in most definitions, such as “emotion”, “describes”, “feel” were removed from the original definitions for the computation of vector representations). We then verified that the 28 selected scales were representative of the initial set of 70 terms by comparing their distributions using a 512-dimensional sentence embedding^69^ (Figure S17A). We also derived a similarity matrix among our 28 scales. 8 factors were extracted that best characterized the semantic structure of our scales (as suggested by visual inspection of the scree plot and the Optimal Coordinate Index) with minimal residual method and rotated with oblimin to allow for potential factor correlations (Figure S17). This showed that our final set of scales were indeed diverse (high dimensional) in terms of their semantic meaning, and that the extracted semantic dimensions bore no resemblance to valence/arousal, arguing that the mere semantic similarity structure of our 28 scales was not a constraint in deriving the low-dimensional solution that we report when the scales were applied to emotion experiences.

#### Stimuli selection

Two types of emotionally evocative stimuli were used: short narratives^18^ and short video clips,^10^ in addition to sampling naturally occurring emotions in participants’ daily lives (Figure 1).

The original set of narratives^18^ consisted of 200 short text paragraphs targeting 20 emotion categories. While the original set described events that happened to another person, we changed them to be in the first person and instructed each participant to imagine themselves in the situation. Each narrative can be represented by a vector of 46 dimensions (valence/arousal, 6 basic emotions and 38 appraisal dimensions) using data from the original paper. We performed principal component analysis (PCA) on randomly sampled subsets of the whole set of narratives (analysis was repeated 100 times for each fixed number of narratives). We found that the number of PCs required to account for 80% of the total variance increased with the number of narratives but reached a plateau around 150 narratives. We therefore determined that 150 narratives were enough to represent the majority of the variance for the whole set. The final set of 150 narratives were selected using a maximum variation sampling procedure. The procedure sampled the narratives by maximizing the sum of Euclidean distances between the narrative vectors. Specifically, the first narrative was randomly selected and subsequent ones were selected so that each new narrative maximized the Euclidean distances from all previously selected ones in the 46-dimensional vector space. The sampling procedure was repeated until the desired sample size was reached. We repeated the whole process for all possible initializations and selected the specific sample with the maximum sum of Euclidean distances.

Similarly for the video clips, we made use of the ratings collected in the original study^10^ and represented each video clip with a vector of 48 dimensions (14 affective and 34 emotion category dimensions, corresponding to the rating scales used in the original paper). A similar PCA procedure as described above was carried out to determine that 1000 video clips contained enough variation. The final set of videos were selected in the following ways. First, given IRB requirements, we deleted a set of 153 extreme videos (depicting, e.g., pornography or torture) from the original set of 2185 videos (corresponding to the videos that are blurred out in the online map of the original study) and then sampled 1000 videos according to the maximum variation procedure as described above. Two videos were still deemed explicitly sexual and were subsequently deleted, which left us with a final set of 998 videos.

The list of the final set of narratives and videos that we used can be found at https://osf.io/7594c/, and interested researchers may contact the authors of the original studies^10^^,^^18^ to get the actual stimuli. To limit session duration, stimuli were divided into 12 separate fixed stimulus sets for administration in 12 sessions). The 150 narratives were randomly divided into 2 fixed sets, each with 75 narratives. Similarly, 998 videos were randomly divided into 10 fixed sets, 9 of them with 100 videos and 1 with 98 videos.

#### Procedure

As mentioned above, the same participant pool was utilized to study emotions evoked by narratives, videos, and in real life. Each wave of the COVID-Dynamic study (16 waves in total, from Apr 2020 to Jan 2021) and each set of the evoked emotion experiments (12 sets in total: 2 sets of narratives and 10 sets of videos, from Nov 2020 to March 2021) were posted as separate studies on Prolific.

##### Emotions sampled in everyday life

We sampled naturally occurring emotions in participants’ daily lives in conjunction with the COVID-Dynamic study. At each wave of data collection, participants were asked to finish an approximately hour-long survey that included assessment on multiple psychological domains using standard and custom questionnaires and performance tasks (see ref.^43^ for the full battery of questionnaires and tasks with frequency of administration).

Participants were asked to rate on 22 scales (a subset of the 28 scales as some were not applicable, collected for every wave since wave 2, 15 waves in total) regarding their current emotion states. Additionally, using free-text responses, in waves 3–16 participants were asked to label their emotion states (minimum of one and maximum of five) and in waves 4–16 they were also asked to explain the causes of these emotions. Note that the content of the free-text responses are available in the interactive UMAP plots that we provide.

More specifically, some scales (*approach, relief, self-relevance*) were removed because they were deemed inapplicable to the emotions experienced in everyday life in our study. For instance, the *relief* scale contrasts how one feels in the end compared to at the beginning of a narrative or video, but this temporal evolution did not apply to the real-life emotions that we were sampling. The other scales (*scalability, and generalizability over stimuli and behavior*) were omitted because we felt they were too complicated to rate, as the real-life emotion ratings were collected as part of an hour-long survey which already poses considerable cognitive and time demands.

##### Evoked emotion experiments

Participants first signed up for the studies on Prolific and were then directed to Qualtrics for informed consent and brief surveys. We posted 12 individual studies on Prolific with 12 different sets of stimuli (2 sets of narratives and 10 sets of video clips). Subsequently, participants were randomly assigned to rate the stimuli on one of 28 affective scales and were directed to Pavlovia (https://pavlovia.org) where the rating experiment was hosted.

The narrative rating experiment consisted of evaluating emotions evoked by 75 narratives on the assigned scale. In the practice trial, participants were shown an example narrative and were asked to move the slider to the middle of the scale (which would be 4 for a scale from 1 to 7) as close as possible. The scale would appear below the narrative once the participants clicked the ‘finished’ button. This was designed to get accurate response time that’s not contaminated by the time spent on reading the narratives. There would be a warning message for clicking the ‘finished’ button too soon within 5 s of the start of the trial. If no rating was made after 50 s, there would be a message to urge for a response or to contact the researcher for assistance. The experiment would end if the participant failed to proceed after 5 min. In the main trials, narratives were presented in random order. Similarly as in the practice trial, participants would read the narrative, click the ‘finished’ button, and then move the slider to rate. Messages would be shown if clicking the ‘finished’ button too soon (within 5 s), or if responding too slow (after 50 s). The trial would be timed out if no response was made within 60 s. And the experiment would end if approximately 10% (8) trials were skipped. In the retest trials where a random set of 8 stimuli previously shown in the main trials would be presented again, participants were asked to rate them again and to also provide the best emotion labels as free text responses which are available in the interactive UMAP plots that we provide. Similarly as before, slow responses would be warned and timed out if necessary.

The video rating task consisted of evaluating about 100 video clips on the assigned scale. In the practice trial, participants were shown an example video (which could be replayed) and were asked to move the slider to the middle of the scale (which would be 4 for a scale from 1 to 7) as close as possible. Once the participants clicked the ‘finished’ button, the scale would appear below and video would be stopped (no replay allowed at this point). This was designed to get accurate response time that’s not contaminated by the time spent on watching the videos. There would be a warning message for clicking the ‘finished’ button too soon within the duration of the video (t) since the start of the trial. If no rating was made after 2t + 50 s, there would be a message to urge for a response or to contact the researcher for assistance. The experiment would end if the participant failed to proceed after 2t + 5 min. In the main trials, videos were presented in random order. Similarly as in the practice trial, participants would watch the video, click the ‘finished’ button and then move the slider to rate. Messages would be shown if clicking the ‘finished’ button too soon (within t), or if responding too slowly (after 2t +50 s). The trial would be timed out if no response was made within 2t +60 s, and the experiment would end if approximately 10% (10) trials were skipped. In the retest trials where a random set of 8 stimuli previously shown in the main trials would be presented again, participants were asked to rate them again and to also provide the best emotion labels as free text responses which are available in the interactive UMAP plots that we provide. Similarly as before, slow responses would be warned and timed out if necessary.

Participants were allowed to finish multiple sessions with different sets of stimuli as they wished, but were not allowed to participate more than once in the same session. We didn’t collect complete data (ratings on all of the scales for all of the stimuli) from every participant as the amount of testing time required would have been around 20 h per participant for narratives alone, and 100 h for videos.

#### Evaluation of scale quality

For each evoked emotion experiment session, we had a small number of retest trials where a random set of 8 stimuli previously shown in the main trials were rated again, thus allowing us to assess within-subject test-retest reliability (using Pearson correlation).

In addition, we also utilized the evoked emotion experiment data to assess between-subject consensus using split-half reliability. Specifically, for participants who rated the same set of stimuli on the same scale (around 20), we randomly split their data into two-halves, and correlated the average ratings derived from the two-halves. Since the split was random, the process was repeated for 50 times to get a more reliable estimate.

Based on the quality metrics described above, we decided to exclude five scales of low quality (*self_relevance*, describing the level of relevance an emotion has to one’s life; *intrinsic_extrinsic*, describing whether an emotion is a reflection of oneself or the surrounding situation; *remembering*, describing the degree to which an emotion involves remembering past events; *mental_bodily*, describing the extent to which an emotion is experienced in the mind or in the body; *future*, describing the degree to which an emotion involves anticipation of future events, resulting in a final set of 23 scales for the evoked emotions and 18 scales for the real-life emotions.

#### Representational similarity analysis (RSA)

RSA provides a framework for comparing the similarity structure across scales derived from different stimulus types.^70^ For the evoked emotion experiments, raw ratings were averaged across participants who rated the same set of stimuli on the same scale to derive average ratings on the 23 rating scales for each of the 150 narratives and 998 videos. For real-life emotions from the COVID-Dynamic study, since emotions for each individual were idiosyncratic, raw ratings were used without averaging. Each of the 12861 real-life emotion experience was described by raw ratings on the 18 rating scales. Average ratings for evoked emotions and raw ratings for real-life emotions as described above were used for the majority of our analyses (RSA, EFA, UMAP, and clustering), unless noted otherwise. We then calculated the scale-by-scale Pearson correlation matrices for emotions evoked by narratives, for emotions evoked by videos and for real-life emotions. To assess how similar the correlation matrices were across stimulus types, we calculated second-order similarity (Pearson correlation coefficients were transformed to normalize their distribution using a Fisher r-to-z transformation before computing secondary-order similarity). Because the correlation matrices are triangle-symmetric, we vectorized the lower triangle of each matrix and calculated Spearman rank-order correlations for each pair of matrices.

#### Exploratory factor analysis (EFA)

We used exploratory factor analysis to gain further insights into the fundamental structure underlying emotion experiences. To determine the optimal number of factors to retain, we took into consideration standard methods based on finding inflection points in the scree plot, results from an empirical cross validation approach where data were randomly split into two-halves with EFA applied to one-half and then confirmatory factor analysis (CFA) applied to the other half to assess the fit of different factor structures, results from the robustness assessments where the number of stimuli and scales were systematically decimated, and the interpretability of the factor loadings.

Once the number of factors to retain was determined, exploratory factor analysis was performed to extract the factors using the minimal residual method, and the solutions were rotated with oblimin rotation for interpretability. The Tenberge method was used to obtain oblique factors scores (using the “‘fa” function in the “psych” package in R).

##### Determining the optimal number of factors

A number of standard statistical methods have been proposed for determining the appropriate number of factors to retain, but no single method is considered to be optimal.^71^ In the hope of finding converging evidence, we included the following methods which are commonly used.

Parallel analysis compares the eigenvalues of the actual data with eigenvalues of random data with the same size and only retains factors that are not due to chance.^72^ Both the optimal coordinate (OC) and the acceleration factor (AF) attempt to provide non-graphical solutions to the scree plot.^73^ OC measures the gradients associated with eigenvalues and their preceding coordinates and finds the elbow based on a series of linear extrapolations. AF tries to identify where the slope of the curve changes most abruptly. The Very Simple Structure (VSS) simplifies the pattern matrix by only keeping the greatest loadings for each item and examines how well the original correlation matrix is reproduced.^74^ Velicer’s Minimum Average Partial test tries to identify factors that represent systematic variances, as opposed to residual or error variance.^75^ Empirical BIC evaluates models with different numbers of factors, taking both the model fit and the parsimony into account.

Parallel analysis, the acceleration factor and the optimal coordinate were computed using the nScree function in the “nFactors” package in R. Very Simple Structure, Empirical BIC and Velicer’s MAP were computed using the nfactors function in the “psych” package.

In addition to the standard statistical procedures described above, we also used a cross validation procedure for determining the number of factors to retain (tested a reasonable range of *n* = 1 to 8 factors). More specifically, for each n, data were randomly split into two-halves (repeated for 20 iterations). EFA was applied to the first half of the data which would result in a factor loading matrix. We then assigned each item to a factor if the absolute loading was higher than a cutoff value of 0.2, and then fitted a CFA model to the other half of the data. To evaluate the performance of different factor solutions, we derived the percentage of explained variance from the EFA and root-mean-square error of approximation (RMSEA) fit index from the CFA.

##### Assessing the robustness of factor solutions

We quantified the robustness of the factor solutions both across different numbers of stimuli and across different numbers of scales.

To test the robustness of our solution against the number of stimuli, we systematically reduced the number of stimuli and computed factor congruences between factors derived using the reduced set and the original set. For the narrative rating data, we started with the full set of 150 narratives, and then removed 5 random narratives (20 randomizations) at each step, until 5 narratives were left at the last step. At each step, we used the new aggregated ratings for EFA and calculated Tucker indices of factor congruence for all sub-datasets (with orthogonal Procrustes rotation). Video rating data were assessed in a similar way where we started with 998 videos, removed 25 (20 randomizations) at each step until 23 videos were left at the last step. For real-life emotions, we started with 12861 instances, and removed 250 (20 randomizations) at each step until 111 instances were left at the last step.

To test the robustness of our solution against the number of scales, we systematically reduced the number of scales and quantified the relatedness of the original factors from the full set and the ones from the subset by correlating the factor scores. The order of removal was determined based on the redundancy of each scale with the rest of the scales. More specifically, we started with the full correlation matrix across all scales after excluding scales of low quality (23 by 23, the average of the two matrices using narrative and video rating data). We quantified the global redundancy of each scale by computing the means of each row (or column), that is, the means of correlations of a scale with all the rest of the scales. The scale with the highest mean correlation and thus the highest redundancy was removed and the correlation matrix was updated. We repeated the same process with the updated matrices until two scales were left. After determining the order of removal of scales, we removed scales one by one as specified and reperformed EFA to extract the same number of factors as before until 5 scales were left. We then quantified the relatedness of the original factors from the full set and the ones from the subset by correlating the corresponding factor scores.

#### Principal components analysis (PCA)

PCA is a statistical technique that reduces the dimensionality of a dataset by linearly transforming the original data into a new coordinate system described by the principal components that maximally preserve the variation. The “principal” function in the “psych” package in R was used to extract the principal components, solutions were rotated with varimax for interpretability.

#### Autoencoder

We used autoencoders (a type of artificial neural networks with minimal assumptions) with cross-validation to compare different factor structures.^76^ Different autoencoders with one hidden layer that differed in the number of units (range from 1 to 8, corresponding to the number of underlying dimensions) were constructed. The input layer and output layer were the same for all models, all layers were densely connected. We trained these different models on half of the data (aggregated ratings across half of the participants) and tested on the other held-out half (repeated for 10 iterations). Both linear and nonlinear activation functions were explored for completeness, but the results were qualitatively similar.

#### Principal preserved component analysis (PPCA)

PPCA is a statistical technique that examines the cross-covariance between two datasets and derives one set of latent variables (principal preserved components, PPCs) that are common to both datasets.^45^ It reduces to PCA when the two datasets are identical. For our implementation, we followed the leave-one-out-cross-validation procedure as described in^10^ and adapted the code shared in.^77^ We didn’t follow the leave-one-rater-out procedure in^77^ because complete data (ratings on all of the scales for all of the stimuli) was not collected from every participant. Note that as repeated measures for each rating was required, this analysis was limited to emotions evoked by narratives and videos only.

#### Uniform Manifold Approximation and Projection (UMAP)

UMAP is a nonlinear dimensionality reduction technique that learns the manifold structure and finds a low dimensional embedding that attempts to preserve as much as possible of the neighborhood relations among data points.^78^ It was used to reduce the original high dimensional space (described by 23 scales for emotions evoked by narratives and videos, and 18 scales for real-life emotions) to a two-dimensional space to visualize the distribution of emotion experiences.

To further elucidate the structure, several color-coding schemes were used. For emotions evoked by narratives and videos, both the categorical labels from the original studies and factor scores were used. Each narrative was associated with one out of 20 intended emotion categories. For videos, we simplified the original data by assigning a single dominant emotion category out of the 34 categories with the highest percentage of selection across participants for each video. Note that four categories (contempt, disappointment, envy, and guilt) were never selected as dominant emotions for any of the videos. For real-life emotions, only factor scores were used.

The most important parameter of UMAP is the size of the local neighborhood, which controls the tradeoff between preserving global and local structure. Specifically, larger values lead to a better preservation of global structure at the expense of local structure preservation. We used a cross-validation procedure to choose this parameter with the goal of preserving both global and local structures. Two metrics were used to evaluate the preservation of local and global structure respectively: trustworthiness, which is based on the change of nearest neighbors^79^ and the Spearman rank correlation of the pairwise Euclidean distances. Data was randomly split into two-halves (repeated 10 times) where one-half was used for training and the other half for testing for a range of neighborhood sizes. The optimal size of the local neighborhood was determined to be 10, 15, and 25 for narrative-evoked, video-evoked and real-life emotions respectively (Figure S2).

#### Clustering

To probe a hierarchy of clusters, we carried out hierarchical agglomerative clustering (HAC, implemented using sklearn.cluster.AgglomerativeClustering in Python) which is a bottom-up approach where each observation starts in its own cluster, and similar clusters are successively merged. Euclidean distance and variance-minimizing Ward linkage were used.^80^ Since all ratings on the scales were in the same range of 1–7, and the actual distributions of each scale contained intrinsic information on how informative the scale was at describing emotion experiences, no transformations were performed to force the same variance across scales.

Selection of instances belonging to the six basic emotion categories were performed with the following criteria. Emotions were described as a 6-dimensional vector corresponding to the ratings on the six basic emotion scales. Thus, a perfect instance of a basic emotion category would be rated as 7 (the highest possible rating) on the corresponding scale and 1 (the lowest possible rating) on all other scales. First, a rating higher than 6 for the corresponding scale was required for an emotion to be considered a good candidate for a given basic emotion category. Second, the Euclidean distance between an emotion and the perfect example should be as small as possible for relatively good specificity. We selected 5 instances (whenever possible) for each basic emotion category for the three stimulus types.

For emotions evoked by narratives and videos, we had the additional information on the intended emotion categories from the original studies and thus asked whether we could re-discover the intended categories as clusters using K-means clustering if the number of clusters were pre-specified. K-means clustering (implemented using sklearn.cluster.KMeans in Python) is a centroid-based clustering algorithm that tries to find the k clusters (as specified) that minimize the inertia, that is, the within-cluster sum-of-squares.^81^

To assess the agreement between the intended labels and the clustering results, we used the following evaluation metrics. The adjusted Rand index (calculated using sklearn.metrics.adjusted_rand_score) is a version of the Rand index corrected for chance, ranging from −1 to 1 with higher values indicating better match.^82^ The unadjusted Rand index indicates how many pairs are in agreement between the clustering result and true labels out of the total number of pairs.^83^ The adjusted mutual information score (calculated using sklearn.metrics.adjusted_mutual_info_score) is a version of the mutual information corrected for chance with higher values indicating better match. The mutual information measures how much information is shared between the clustering result and the ground truth.^84^ A contingency matrix (calculated using sklearn.metrics.cluster.contingency_matrix) can be used to visualize the agreement for every true/predicted cluster pair, allowing one to examine the spread of each true cluster across predicted clusters and vice versa.

### Quantification and statistical analysis

To test the distribution of pairwise distances in the original high dimensional space for unimodality, the Hartigan’s dip test was used (implemented using the “diptest” package). A large value of the dip statistic and a small *p*-value indicates a substantial deviation from a unimodal distribution, thus rejecting the null hypothesis of unimodality.

For representational similarity analysis, to assess the relatedness of matrices (that is, the scale-by-scale Pearson correlation matrices for emotions evoked by narratives, for emotions evoked by videos and for real-life emotions), we followed a randomization procedure^70^: a distribution of 10,000 correlations was obtained by permuting the rows and columns of one of the two correlation matrices and the actual correlation was then compared to this distribution to derive the empirical *p*-value.

For principal preserved component analysis, our implementation involved the following steps. 1) Half of the ratings for each stimulus were randomly assigned into two sets, ratings were averaged within each set. 2) In a leave-one-out-cross-validation procedure, one stimulus was held out at a time and PPCA was performed on the remainder of the stimuli between the two sets to extract PPCs. Then we projected the held-out stimulus onto the components. 3) Component scores for held-out stimuli were concatenated for each set and correlated across the two sets (partialling out each previous component). 4) Steps 1 to 3 were repeated 20 times. We tested if these correlations were consistently positive for each component using a one-tailed Wilcoxon signed-rank test with Bonferroni correction (significance level of 0.05).

For our analysis of individual differences, each experiment session involved rating roughly 75 narratives or 100 videos on a single scale (23 remaining after exclusion) for emotions evoked by narratives or videos. We first centered each stimulus on its average rating, from all ratings of that stimulus on that scale across participants, and then computed the mean rating of a session for each participant. Eliminating the effect of stimuli allowed us to compare each participant’s mean ratings across stimuli with different raw means. This also served as a general baseline that represented the rating bias of each participant. For emotions sampled in daily life, we first computed the mean ratings across all waves on all scales (18 remaining after exclusion) for each participant. Note that this analysis was restricted to participants with complete data from all 15 waves (a sample size of 441, reduced from the original 1000 after exclusion) so as to avoid any possible temporal biases. In one version of the analysis, the initial within-subject mean ratings across waves were corrected for the baseline rating bias as obtained from narrative/video ratings whenever available. We then associated participant’s mean ratings on each of the scales (both evoked and those in real life) with demographic and psychological variables (averaged across multiple time points if repeated assessments were available, collected as part of the COVID-Dynamic study, see Table S3 for the details of the measures) using Pearson’s correlations for continuous variables and Welch’s t test for categorical variables (implemented using the “pearsonr” and “ttest_ind” functions in the “SciPy” package).

All statistical analyses were run in Python 3.7.6 using scripts that implemented either the standard tests or custom algorithms as described above.
