# Supplementary material for: A shared structure for emotion experiences from narratives, videos, and everyday life
Source: iScience. 2024 Jun 24;27(7):110378. doi: 10.1016/j.isci.2024.110378 (PMC11296042; doi:10.1016/j.isci.2024.110378)
Supplement: Document S1. Figures S1–S18 and Tables S1–S4 [file mmc1.pdf]

iScience, Volume *27*

Supplemental information

A shared structure for emotion experiences from narratives, videos, and everyday life

Yanting Han, The COVID-Dynamic Team, and Ralph Adolphs

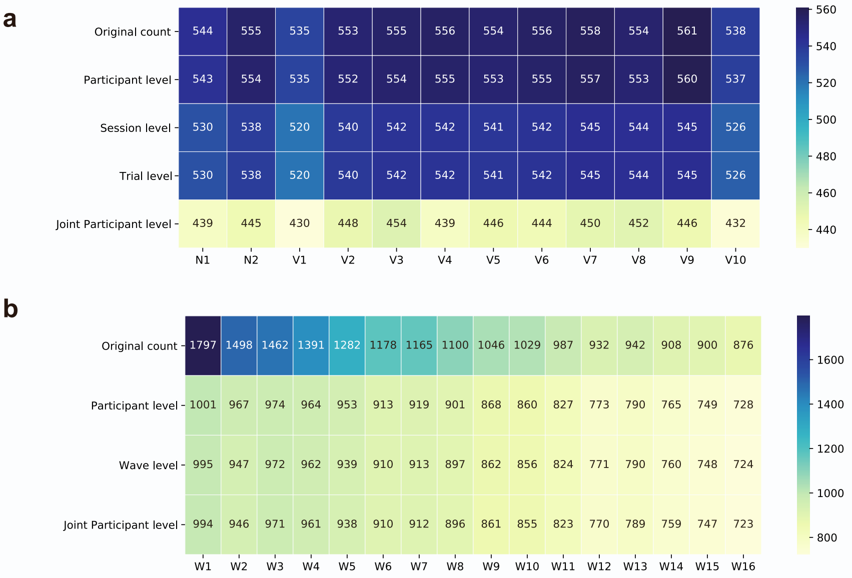

Fig. S1. Data exclusion, Related to STAR Methods. (a) Evoked emotion experiments: the number of remaining participants after each level of exclusion (each row) for different stimulus sets (each column: narrative: N1, N2; video: V1 to V10). (b) Real-life emotions: the number of remaining participants after each level of exclusion (each row) for different waves (each column, from wave 1 to wave 16).

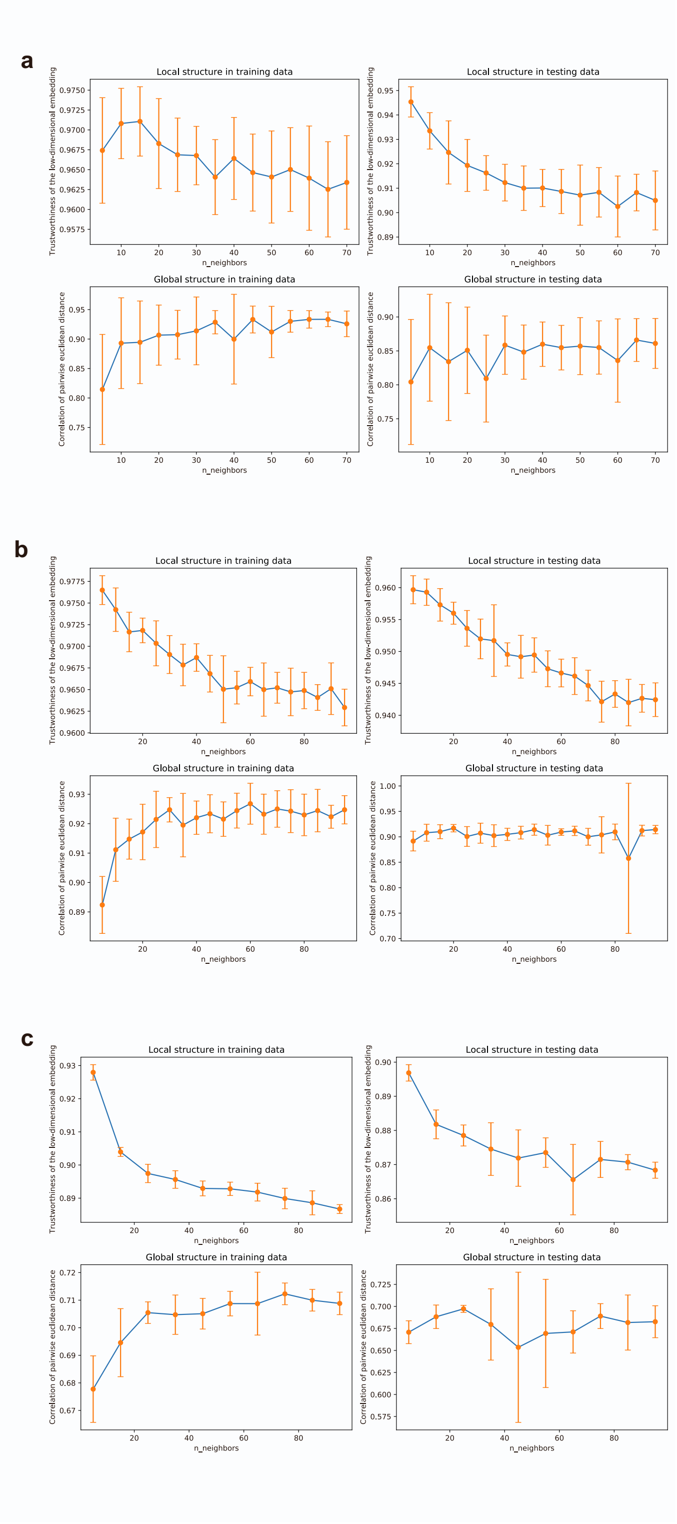

Fig. S2. Parameter selection for UMAP, Related to STAR Methods. The means (dots) and standard deviations (error bars, n = 10 iterations) of trustworthiness (upper) and rank correlation of pairwise distances (lower) of the training data and testing data (left to right) for different sizes of the local neighborhood for (a) emotions evoked by narratives, (b) emotions evoked by videos, and (c) real-life emotions.

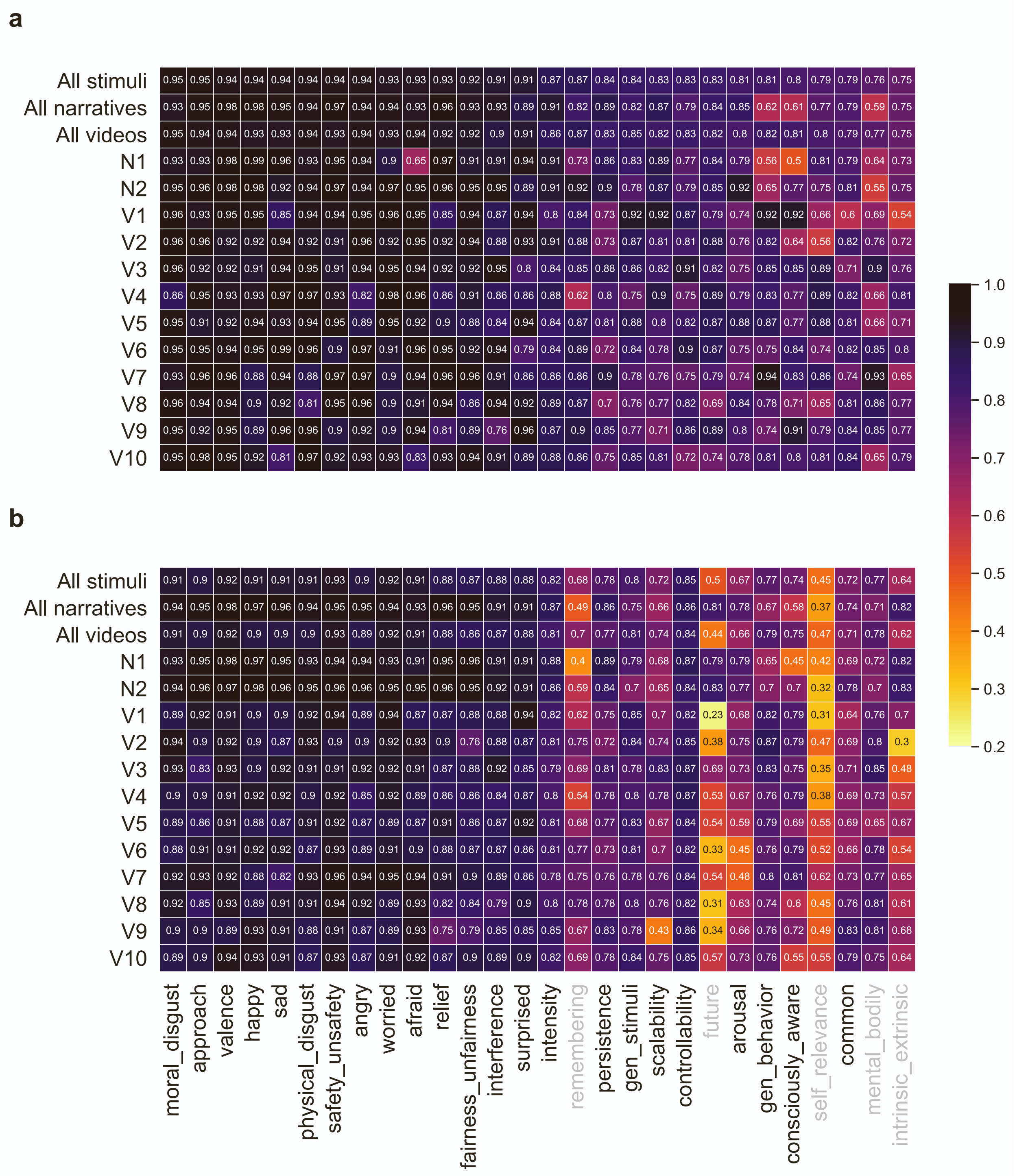

Fig. S3. Evaluation of scale quality, Related to Figure 2. (a) median test-retest reliability and, (b) median split-half reliability for each scale calculated using different data (all experiment sessions, all narrative sessions, all video sessions, and individual sessions alone). Scales were sorted based on median test-retest reliability across all stimuli. Five scales (in grey) were excluded because of low quality.

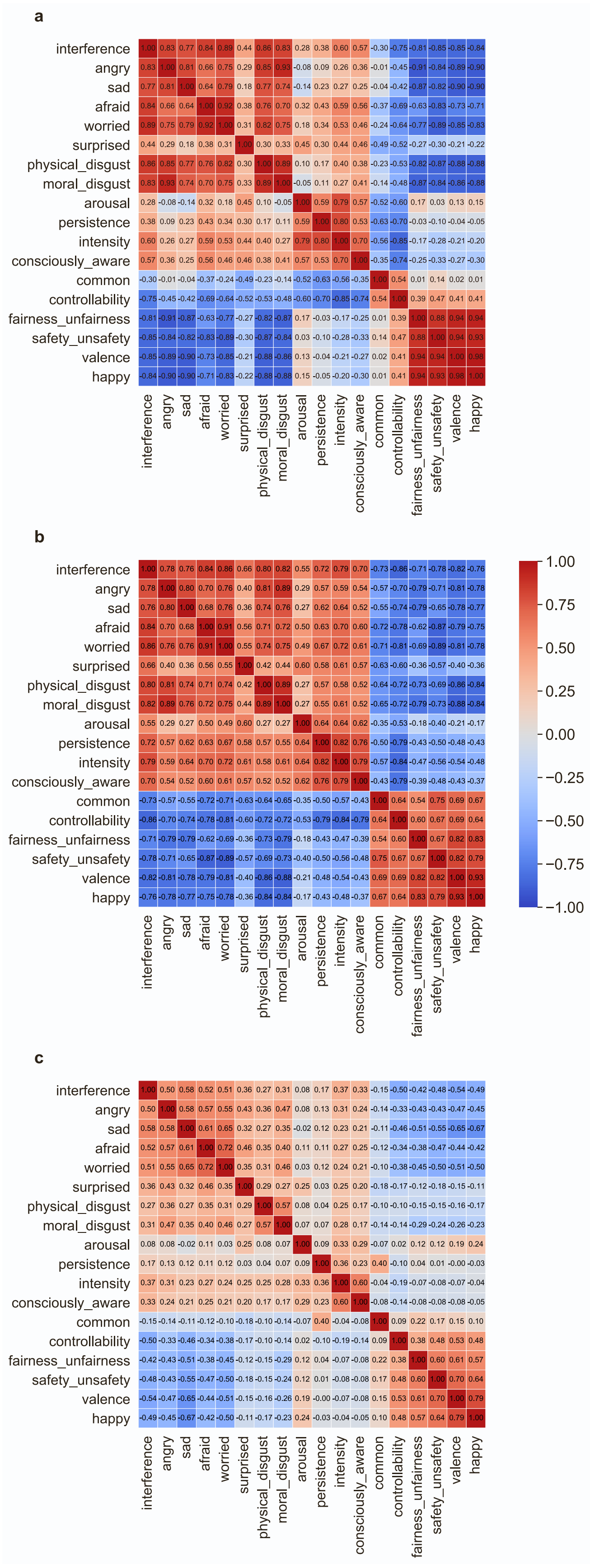
a
b
c
interference
angry
sad
afraid
worried
surprised
physical_disgust
moral_disgust
arousal
persistence
intensity
consciously_aware
common
controllability
fairness_unfairness
safety_unsafety
valence
happy
1.00
0.75
0.50
0.25
0.00
−0.25
−0.50
−0.75
−1.00

Fig. S4. Representational structures across stimulus domains, Related to Figure 3. Correlation matrices across 18 shared scales for (a) emotions evoked by narratives, (b) emotions evoked by videos, and (c) real-life emotions. All matrices were sorted using hierarchical clustering applied to real-life emotions for easier comparison.

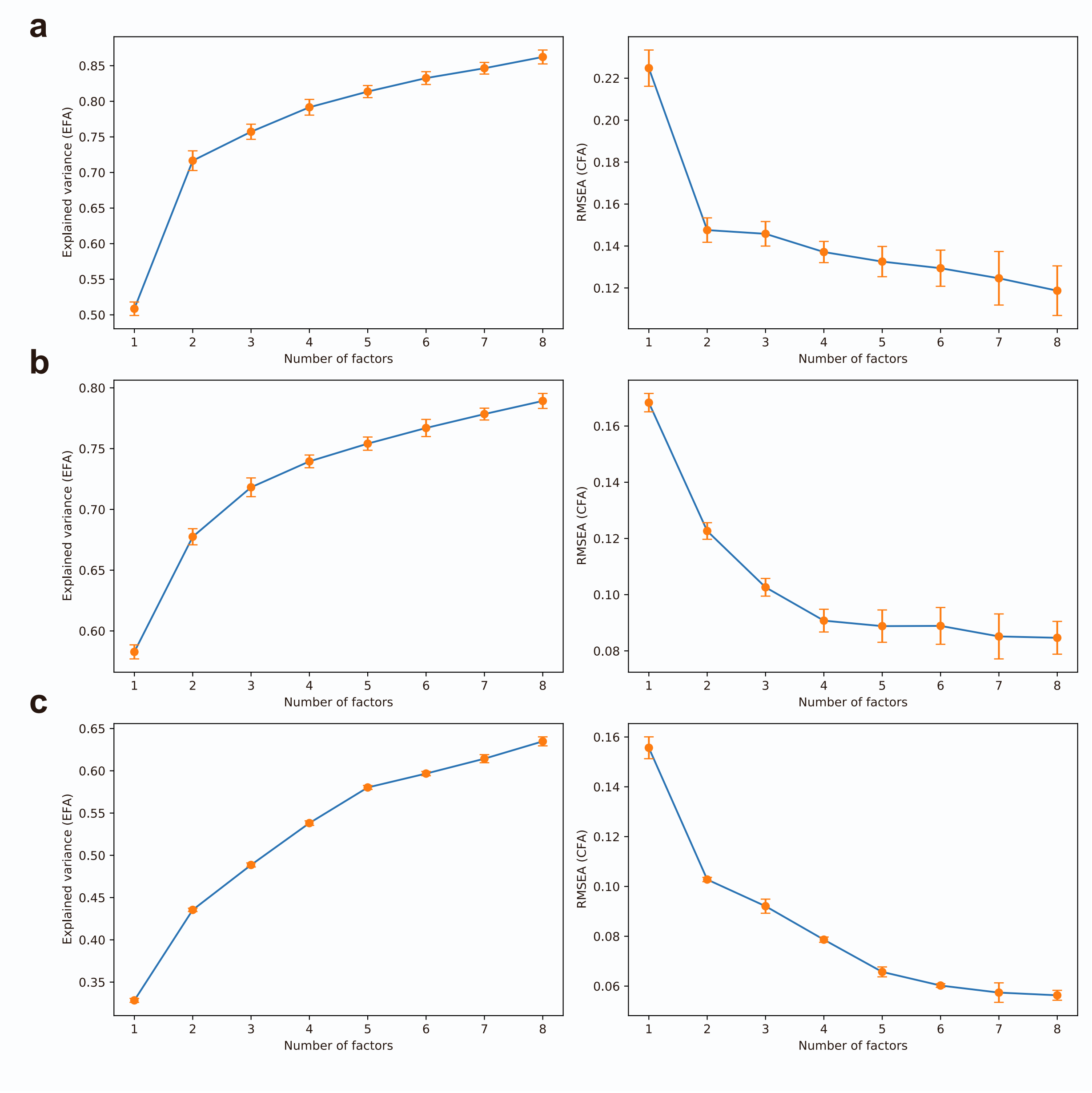

Fig. S5. Dimensionality analysis with cross-validation, Related to STAR Methods and Figure 4. The means (dots) and standard deviations (error bars, n = 20 iterations) of explained variance from the EFA on training data on the left, and root mean square error of approximation (RMSEA) fit index from the CFA on testing data on the right for (a) emotions evoked by narratives, (b) emotions evoked by videos, and (c) real-life emotions.

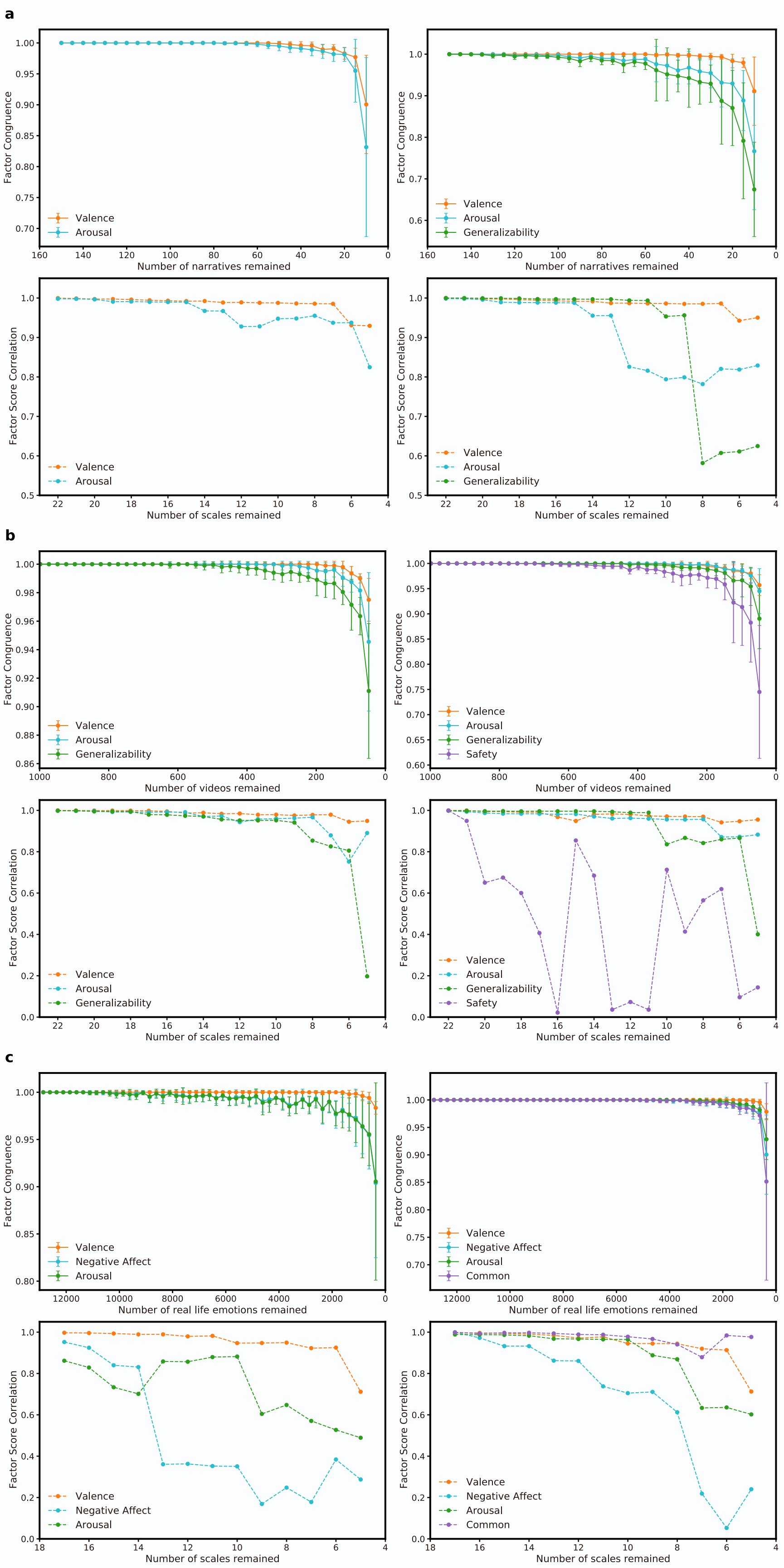
a
Factor Congruence
Number of narratives remained
Valence
Arousal
Generalizability
Factor Score Correlation
Number of scales remained
b
Number of videos remained
Safety
c
Number of real life emotions remained
Negative Affect
Common

Fig. S6. Robustness of factor solutions with respect to the number of stimuli and number of scales (Upper: Points indicate the means and error bars indicate standard deviations of Tucker indices of factor congruence (with orthogonal Procrustes rotation) between the full set versus subsets of stimuli across 20 iterations, and are color-coded for different factors, Related to STAR Methods and Figure 4. Lower: Pearson's correlations between factor scores from the full set versus subsets of scales, color-coded for different factors) for (a) emotions evoked by narratives: the 2 factor solution on the left and the 3 factor solution on the right, (b) emotions evoked by videos: the 3 factor solution on the left and the 4 factor solution on the right, and (c) real-life emotions: the 3 factor solution on the left and the 4 factor solution on the right.

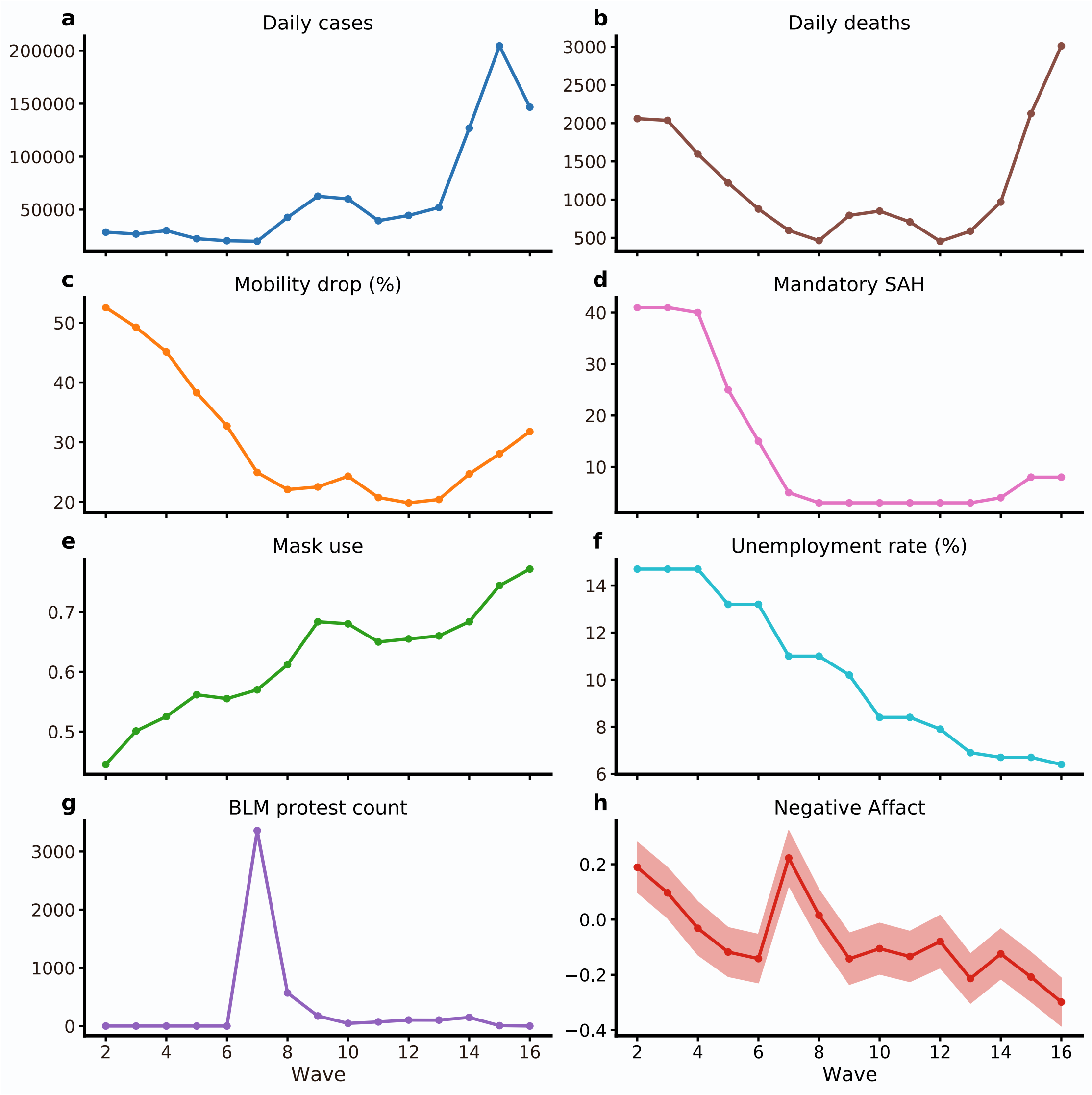

Fig. S7. Temporal trend across waves for (a-g) covid-related metrics at the US national level (a: daily covid cases, b: daily covid deaths, c: percent change in mobility from baseline, d: total number of states with mandatory stay at home restrictions, e: percent of population reporting always wearing a mask when leaving home, f: unemployment rate, and g: anti-racism protest counts), and (h) 'negative affect' factor in real-life emotions, Related to Figure 4. Data sources: https://www.healthdata.org/covid/data-downloads and https://coviddynamic.caltech.edu/data-sharing.

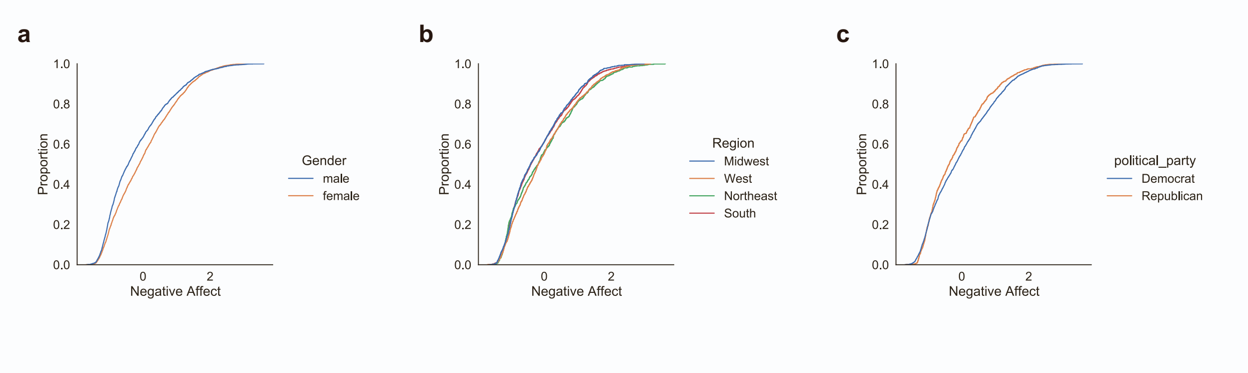

Fig. S8. Individual differences in 'negative affect' factor in real-life emotion experiences, Related to Figure 4. Empirical cumulative distribution function for different groups partitioned based on (a) gender, (b) geographic regions, and (c) political parties.

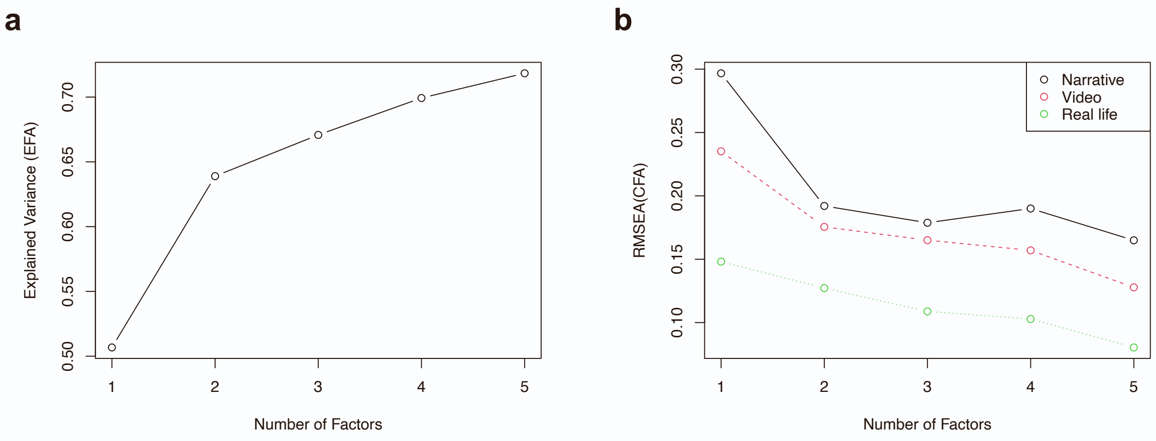

Fig. S9. Dimensionality analysis of the shared structure across stimulus domains, Related to STAR Methods and Figure 4. (a) explained variance from the EFA on the averaged correlation matrix, and (b) root mean square error of approximation (RMSEA) fit index from the CFA on each of the three types of data (indicated by different colors).

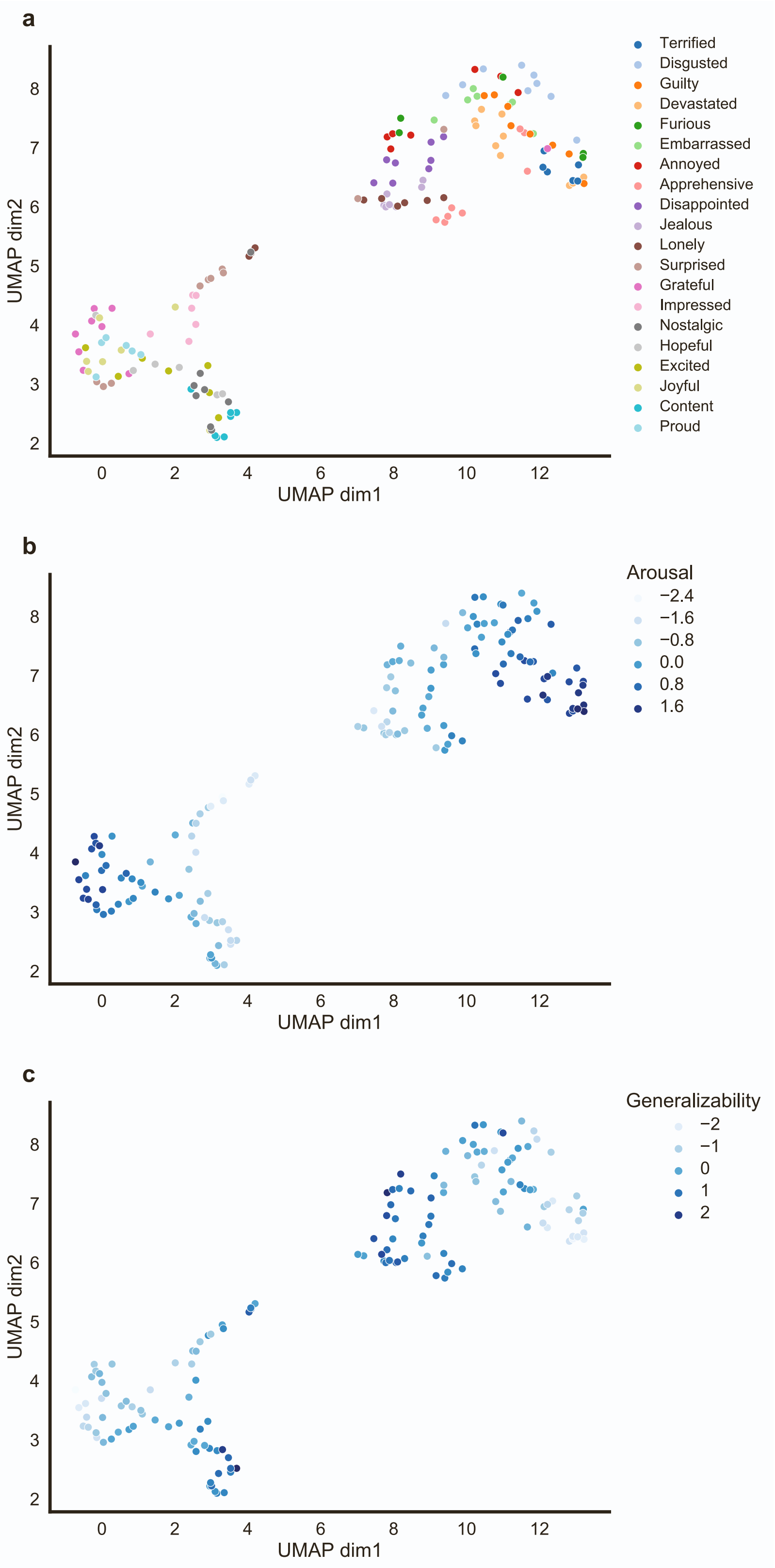

Fig. S10. Distribution of emotion experiences evoked by narratives, Related to Figure 5. UMAP plots color-coded for (a) intended categories, (b) the “arousal” factor, and (c) the “generalizability” factor.

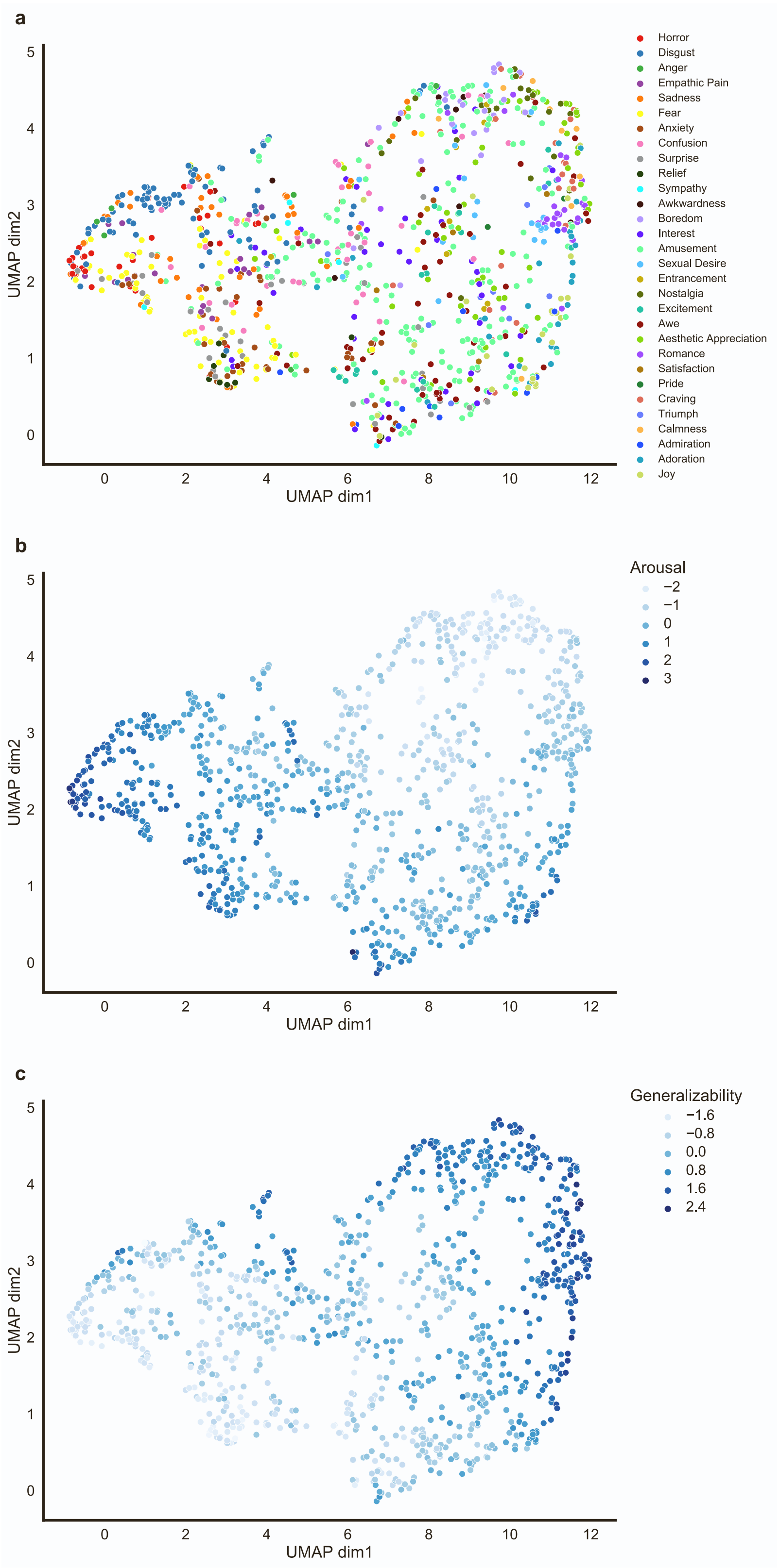

Fig. S11. Distribution of emotion experiences evoked by videos, Related to Figure 5. UMAP plots color-coded for (a) dominant emotion categories, (b) the “arousal” factor, and (c) the “generalizability” factor.

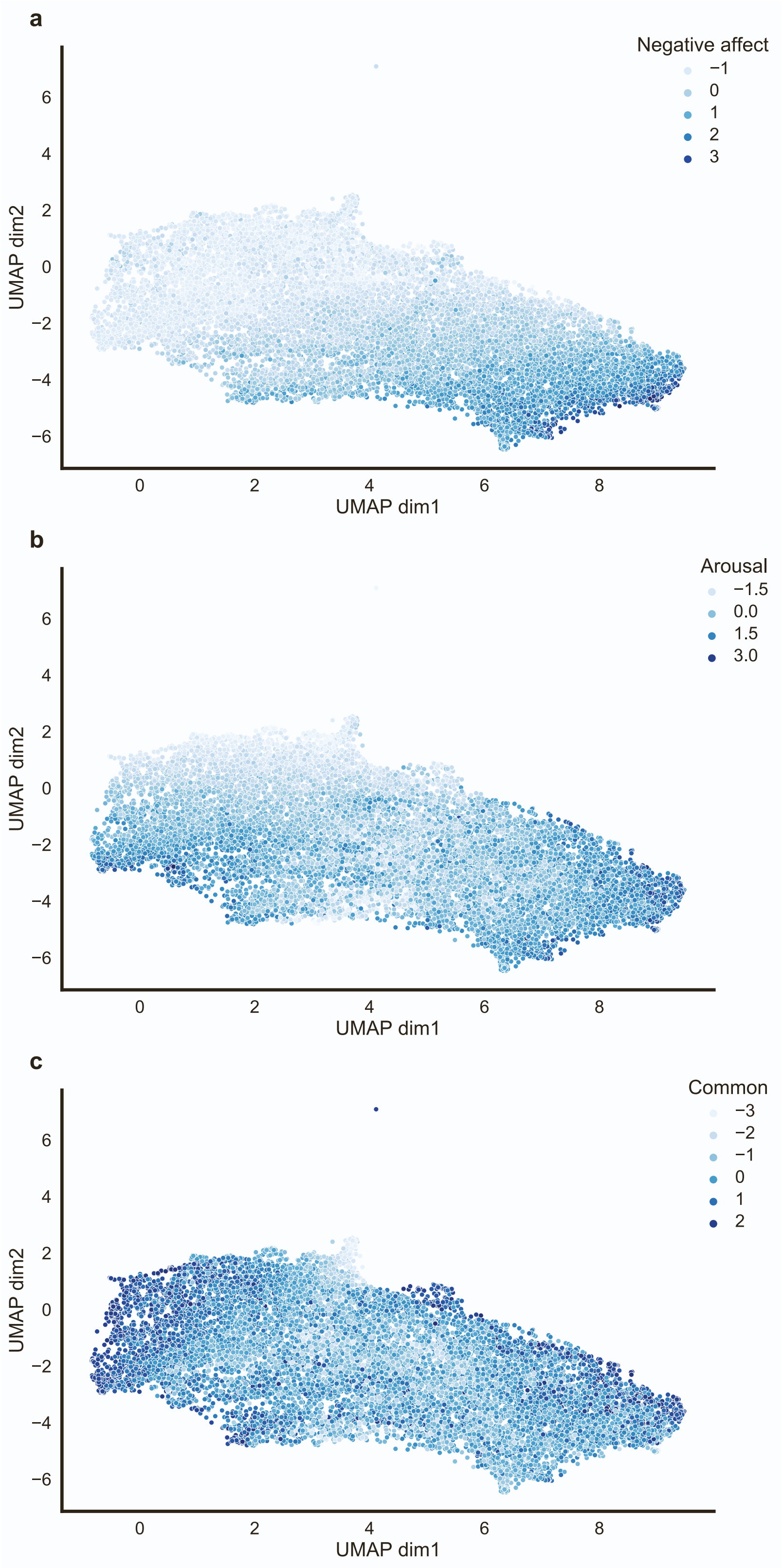

Fig. S12. Distribution of real-life emotion experiences, Related to Figure 5. UMAP plots color-coded for (a) the “negative affect” factor, (b) the “arousal” factor, and (c) the “common” factor.

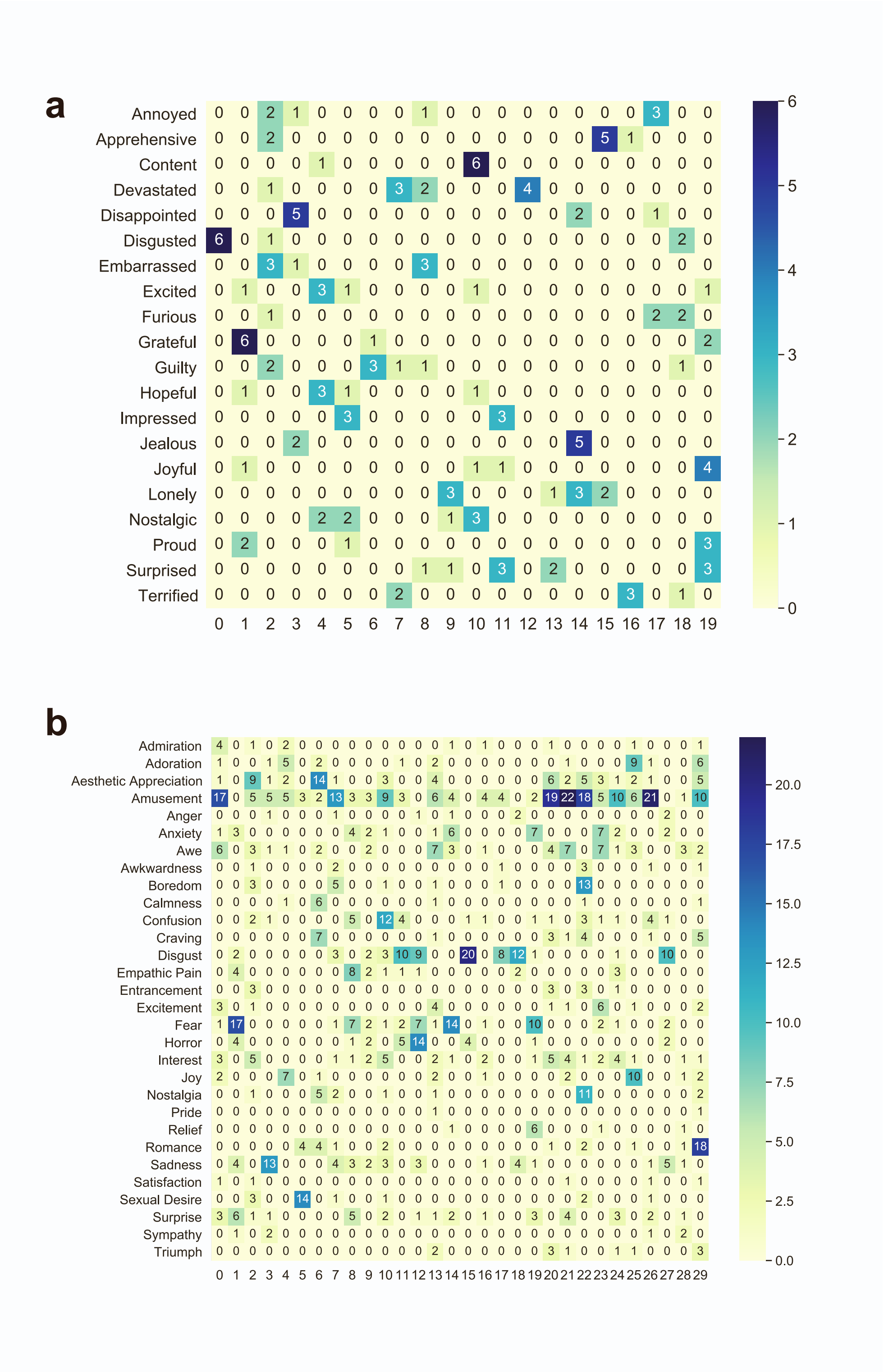

Fig. S13. Recovery of intended categories, Related to Figure 6. (a) Contingency matrix between the 20 discovered categories (columns) and the ones intended (rows) for emotions evoked by narratives, and (b) Contingency matrix between the 30 discovered categories (columns) and the ones intended (rows) for emotions evoked by videos.

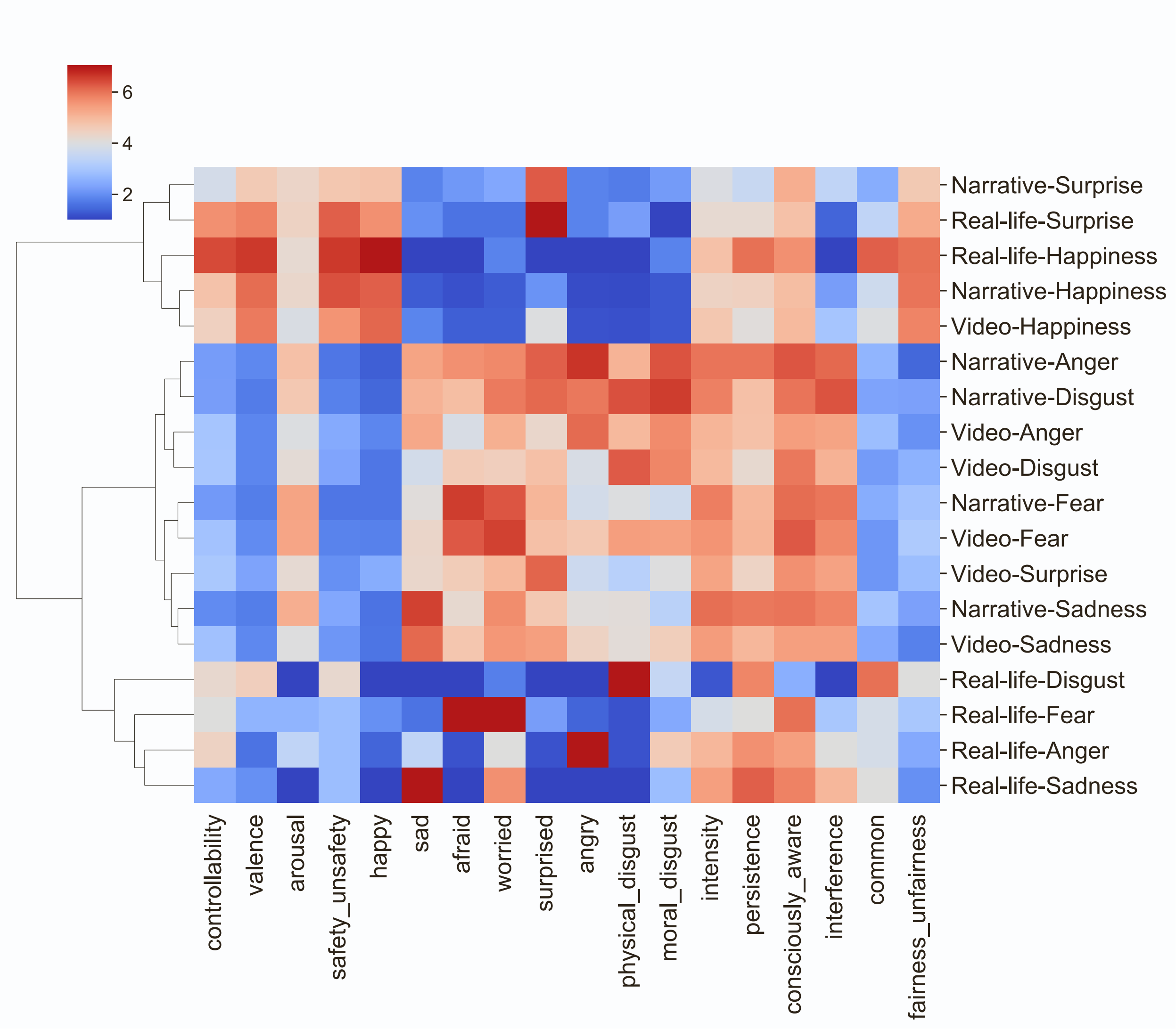

Fig. S14. Hierarchical structure of basic emotions (averaged within each basic emotion category) , Related to Figure 6. Each column indicates the averaged ratings on the 18 scales and each row represents one emotion category of a stimulus type.

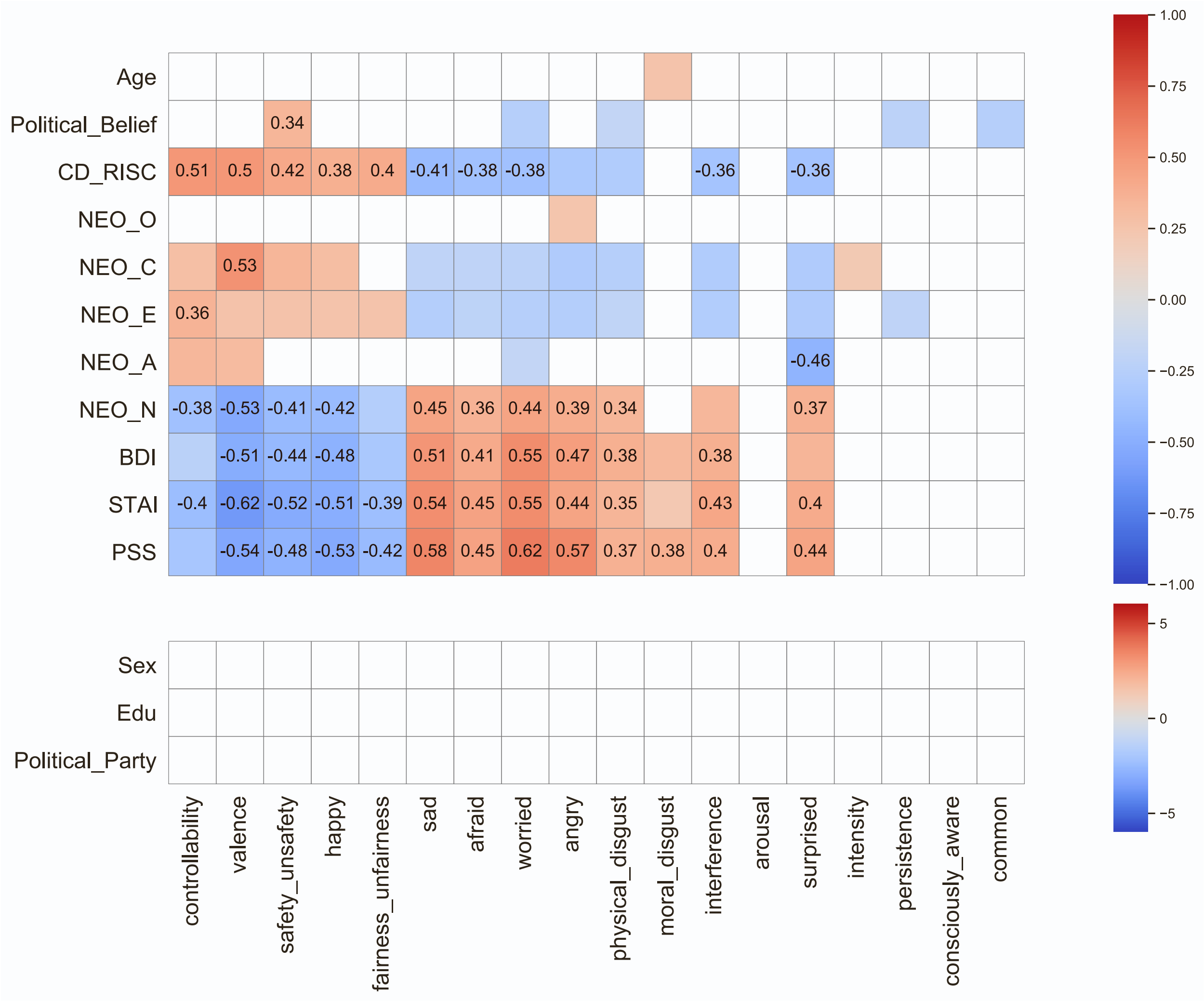

Fig. S15. Individual differences in real-life emotion experiences (corrected for baseline rating biases from narrative/video ratings), Related to Figure 7. Pairwise Pearson's correlations (upper) between ratings and demographic and psychological variables, and Welch's t-test (lower) for means of ratings of different groups (divided based on sex, education and political party; t-statistics for males - females, high - low education, and republicans - democrats respectively). Raw results with $p < 0.05$ are colored (otherwise masked) and Bonferroni corrected results with $p < 0.05$ are annotated with values.

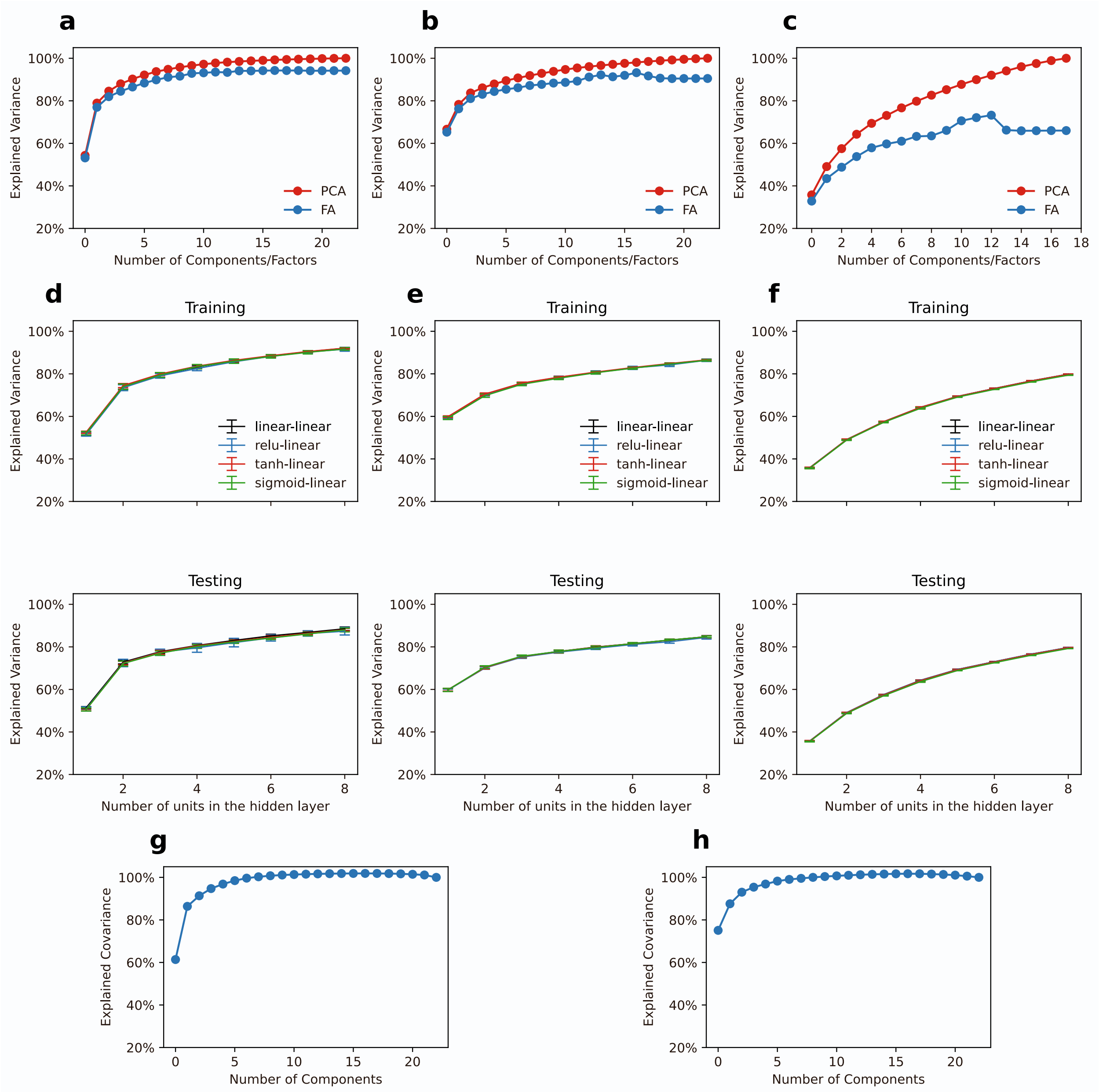

Fig. S16. Dimensionality reduction results, Related to STAR Methods and Figure 4. The percentage of explained variance by EFA and PCA for (a) emotions evoked by narratives, (b) emotions evoked by videos, and (c) real-life emotions. The means (points) and standard deviations (bars) of the explained variance (10 iterations) on the training data and testing data from autoencoders with various numbers of units in the hidden layer for (d) emotions evoked by narratives, © emotions evoked by videos, and (f) real-life emotions (colors indicate different configurations of activation functions in the encoder and decoder layers). The percentage of explained covariance by PPCA for (g) emotions evoked by narratives, (h) emotions evoked by videos.

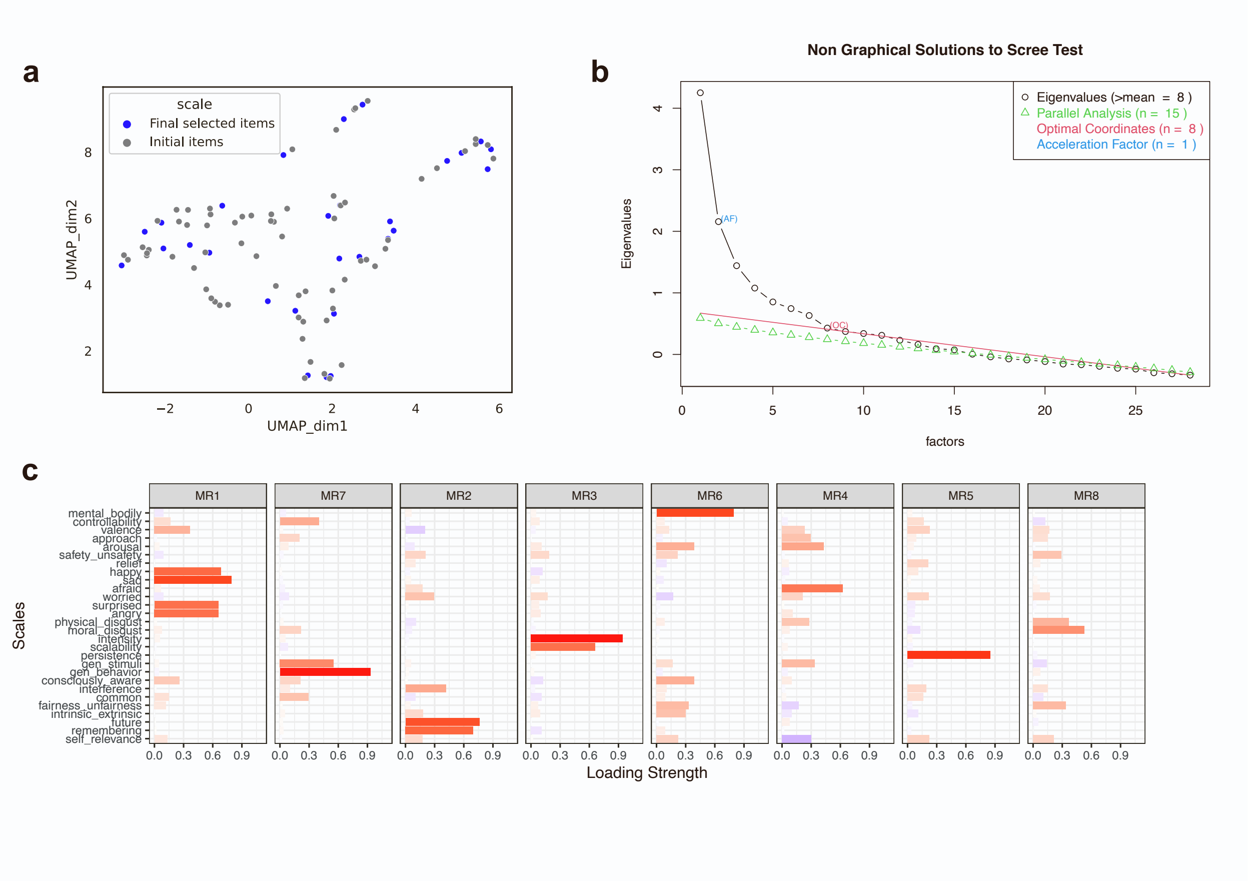

Fig. S17. Dimensionality of the semantics of rating scales, Related to STAR Methods. (a) UMAP plot of rating scales. Blue dots indicate the final set of 28 rating scales and gray dots indicate the initial list of items sampled from literature. (b) Scree plot with parallel analysis, the acceleration factor, and the optimal coordinate. (c) Factor loadings: each column plots the strength of the factor loadings (x-axis, absolute value) across scales (y-axis). Color indicates the sign of the loading (red for positive and blue for negative), more saturated colors for higher absolute values.

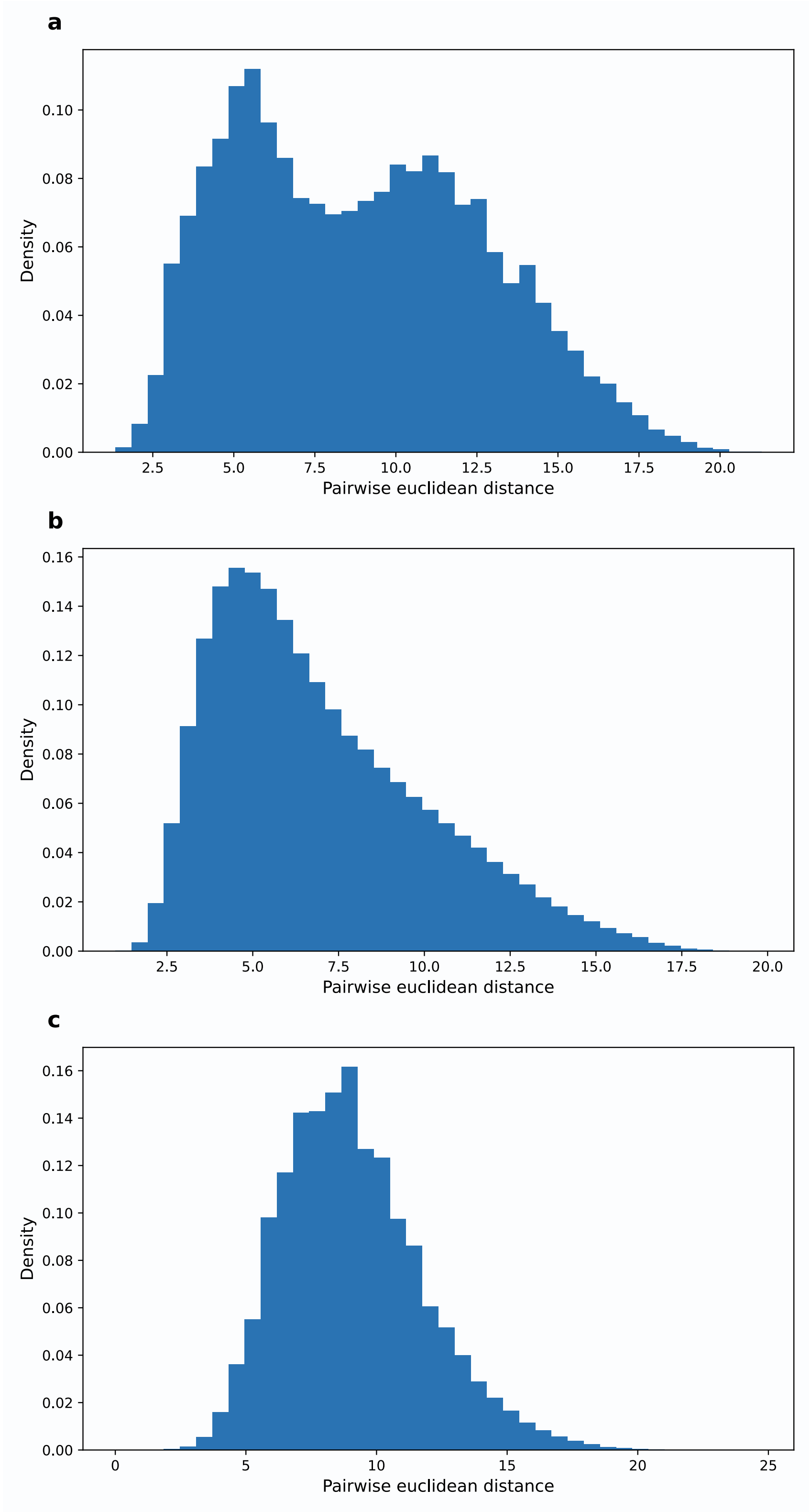

Fig. S18. Histograms of pairwise Euclidean distances in the original high dimensional spaces for (a) emotions evoked by narratives, (b) emotions evoked by videos, and (c) real-life emotions, Related to Figure 5 and Figure 6.

Table S1. Definition of 28 rating scales, with the description of the two ends of the scales and grade level required to understand the definitions, Related to STAR Methods and Figure 1.

Labels	Definitions	Lower_end	Higher_end	Grade level
mental_bodily	this scale describes the extent to which an emotion is experienced in the mind or in the body	experienced mostly in the mind	experienced mostly in the body	7.82
Controllability	this scale describes how much control you have over an emotion	cannot control this emotion at all	this emotion is easy to control	5.86
valence	this scale describes how pleasant or unpleasant an emotion is	very unpleasant	very pleasant	7.19
approach	this scale describes how much an emotion makes you want to approach or avoid	strongly want to avoid	strongly want to approach	5.88
arousal	this scale describes how physically aroused/stimulated an emotion makes you feel	not aroused at all	highly aroused	10.72
safety_unsafety	this scale describes the degree to which an emotion evokes a sense of safety or unsafety	evokes a strong sense of unsafety	evokes a strong sense of safety	9.82
relief	this scale describes how the feeling turns out to be in the end compared to how you felt in the beginning	felt much worse than it was at the beginning	felt much better than it was at the beginning	7.21
happy	this scale describes how happy this emotion makes you feel	not happy at all	very happy	4.83
sad	this scale describes how sad this emotion makes you feel	not sad at all	very sad	3.65
afraid	this scale describes how afraid (which is immediate and directed towards the present stimulus) this emotion makes you feel	not afraid at all	very afraid	11.07

worried	this scale describes how worried (which is more diffused and longer lasting towards a future threat or risk) this emotion makes you feel	not worried at all	very worried	9.8
surprised	this scale describes how surprised this emotion makes you feel	not surprised at all	very surprised	4.83
angry	this scale describes how angry this emotion makes you feel	not angry at all	very angry	4.83
physical_disgust	this scale describes how physically disgusted (towards things like vomit and spoiled food) this emotion makes you feel	not disgusted at all	very disgusted	9.79
moral_disgust	this scale describes how morally/socially disgusted (towards things like acts of violating social norms) this emotion makes you feel	not disgusted at all	very disgusted	12.27
intensity	this scale describes how strong or weak an emotion is	very faint emotion	very strong, intense emotion	3.65
scalability	this scale describes how much an emotion can scale in intensity. If it does not scale, then it always feels equally strong or weak. Otherwise, it can be either strong or weak.	doesn’t scale	can scale	4.8
persistence	this scale describes how long an emotion lasts	emotion is very brief and fleeting	emotion sticks for a long time	3.76
gen_stimuli	this scale describes how many different stimuli can evoke a certain emotion	very rare and specific	very common and often found	10.72
gen_behavior	this scale describes how many different behaviors an emotion can cause	very rare and specific	very common and often found	10.15
consciously_aware	this scale describes how consciously aware you are of an emotion	not aware at all	very aware	8.01

interference	this scale describes the degree to which an emotion disrupts other ongoing activities	not disruptive at all	very disruptive	10.36
common	this scale describes how often you've felt like this	very rare, not often experienced	very common, I experience this emotion on a regular basis	2.34
fairness_unfairness	this scale describes the degree to which an emotion evokes a sense of unfairness or fairness	evokes a strong sense of unfairness	evokes a strong sense of fairness	8.35
intrinsic_extrinsic	this scale describes whether an emotion is primarily a reflection of you (e.g. your personality, your abilities, your past experiences) or a reflection of the surrounding situation (other people, external forces)?	completely intrinsic	completely extrinsic	20.48
future	this scale describes the degree to which an emotion involves anticipation of an event that would or might occur in the future	not related to anticipation of future events at all	totally related to anticipation of future events	11.23
remembering	this scale describes the degree to which an emotion involves remembering events occurred in the past	not related to past events at all	totally related to past events	9.82
self_relevance	this scale describes the level of relevance an emotion has to your life	low relevance	high relevance	6.73

Table S2. Demographic characteristics (means and standard deviations) of all measures for the final sample after exclusion, Related to STAR Methods and Figure 1.

	**All (N = 1000)**	**Female (N=507)**	**Male (N=493)**
Age (in years) (mean; sd)	39.65 (14.22)	40.39 (14.47)	38.89 (13.92)
Education: below Bachelor (n; %)	442 (44.2%)	220 (43.39%)	222 (45.03%)
Education: Bachelor and above (n; %)	558 (55.8%)	287 (56.61%)	271 (54.97%)
CD-RISC (mean; sd)	26.38 (7.3)	25.59 (7.4)	27.18 (7.12)
NEO Openness (mean; sd)	30.75 (6.51)	31.69 (6.47)	29.79 (6.42)
NEO Conscientiousness (mean; sd)	33.34 (7.54)	33.26 (7.38)	33.43 (7.7)
NEO Extraversion (mean; sd)	22.68 (8.26)	22.34 (8.1)	23.04 (8.42)
NEO Agreeableness (mean; sd)	32.75 (6.26)	33.82 (5.89)	31.66 (6.45)
NEO Neuroticism (mean; sd)	20.4 (10.55)	22.18 (10.61)	18.58 (10.19)
BDI (mean; sd)	11.2 (10.12)	12.16 (10.28)	10.21 (9.88)
STAI (mean; sd)	41.82 (12.4)	43.12 (12.65)	40.49 (12.0)
PSS (mean; sd)	15.71 (7.24)	17.01 (7.2)	14.38 (7.03)

Table S3. Demographic and psychological measures for individual differences, Related to STAR Methods and Figure 1.

Age: in years.
Sex: birth sex (male or female).
Education: highest level of education completed (low: below bachelor, high: bachelor and above).
Political Party: political party that one identifies with (Republican or Democrat).
Political Belief: liberal/conservative rating on a seven-point Likert scale (1 strongly liberal to 7 strongly conservative).
CD-RISC: The Connor-Davidson Resilience Scale - 10 Item [1] is a self-report questionnaire of coping responses in the past month.
NEO: The NEO Five-Factor Personality Inventory [2] is a 60-item self-report questionnaire that assesses an individual on five dimensions of personality: openness (NEO_O), conscientiousness (NEO_C), extraversion (NEO_E), agreeableness (NEO_A), and neuroticism (NEO_N).
BDI: Beck Depression Inventory – II [3] is a 21-item self-report questionnaire that examines depressive symptomatology over the past two weeks.
STAI: The State Trait Anxiety Inventory [4] is a 20-item self-report questionnaire that measures state and trait anxiety, only trait one is used here.
PSS: The Perceived Stress Scale [5] is a 10-item self-report questionnaire that measures the extent to which a participant perceives personal life events in the past month as stressful.

Table S4. Contributions by the COVID-Dynamic team (0: no contribution, 1: minimal contribution, 2: substantial contribution, 3: lead contribution), Related to STAR Methods.

	Yanting Han	Ralph Adolphs	Lynn Paul	Uri Maoz	Tessa Rusch	Damian Stanley
Conceptualization: Initial Inception of Article	3	3	0	0	0	0
Conceptualization: Composition and Scope of Article	3	3	0	0	0	0
Data Acquisition	3	1	3	1	3	2
Data Curation, Q/A, Sharing	3	0	3	0	2	1
Data analysis	3	0	0	0	0	0
Funding Acquisition	0	3	0	0	0	1
Methodology: Questionnaire and Task Selection/ Development	3	2	2	1	0	1
Supervision	0	2	0	0	0	0
Writing: Original draft	3	1	0	0	0	0
Writing: Review & Editing	3	3	1	0	0	1
Conceptualization: Initial Inception of CovidDynamic project	3	3	1	3	3	3
Conceptualization: Composition and Scope of CovidDynamic Project	3	1	3	2	0	3

SI References

1. Connor, K.M., and Davidson, J.R. (2003). Development of a new resilience scale: The Connor ‐ Davidson resilience scale (CD ‐ RISC). Depression and anxiety *18*, 76-82.
2. Costa, P.T., and McCrae, R.R. (2008). The revised neo personality inventory (neo-pi-r). The SAGE handbook of personality theory and assessment *2*, 179-198.
3. Beck, A.T., Steer, R.A., and Brown, G.K. (1996). Manual for the beck depression inventory-II. San Antonio, TX: Psychological Corporation *1*, 10.1037.
4. Spielberger, C.D. (1983). State-trait anxiety inventory for adults.
5. Cohen, S., Kamarck, T., and Mermelstein, R. (1983). A global measure of perceived stress. Journal of health and social behavior, 385-396.
